# Enantioselective 5-*exo*-Fluorocyclization of Ene-Oximes

**DOI:** 10.3390/molecules24193464

**Published:** 2019-09-24

**Authors:** Taiki Rouno, Tomoki Niwa, Kousuke Nishibashi, Nobuharu Yamamoto, Hiromichi Egami, Yoshitaka Hamashima

**Affiliations:** School of Pharmaceutical Sciences, University of Shizuoka, 52-1 Yada, Suruga-ku, Shizuoka 422-8526, Japan; s18125@u-shizuoka-ken.ac.jp (T.R.); s18405@u-shizuoka-ken.ac.jp (T.N.); m14085@u-shizuoka-ken.ac.jp (K.N.); m16117@u-shizuoka-ken.ac.jp (N.Y.); hegami@u-shizuoka-ken.ac.jp (H.E.)

**Keywords:** fluorine, asymmetric catalysis, phase-transfer catalysis, oxime

## Abstract

The enantioselective 5-*exo*-fluorocyclization of ene-oxime compounds was demonstrated under phase-transfer catalysis. Although deprotonative fluorinations competed, the chemical yields and the ee values of the desired isoxazoline products were generally moderate to good. The absolute stereochemistry of the major isomer was determined to be *S* by comparison with the literature after transformation of the product to the corresponding iodinated isoxazoline.

## 1. Introduction

Isoxazoline framework has been recognized as a significant substructure of natural and unnatural compounds [1,2], which possess various biological activities including anticancer activity [3,4], FXa inhibitory activity [5], and antiparasitic activity [6,7,8,9]. Therefore, the construction of isoxazoline has been actively studied [10,11,12,13]. Among various methods for isoxazoline synthesis, a difunctionalization-type cyclization of ene-oxime is one of the major strategies. Cyclization reactions with concomitant introduction of a fluoroalkyl group [14,15,16,17], a hydroxyl group [18,19], a thiocyanate group [20], and an iodine group [21] have been reported so far.

Fluorine chemistry has contributed to pharmaceutical and agrochemical sciences, because an introduction of fluorine atom(s) at an appropriate position often improves the property of the parent compounds in terms of metabolic stability, lipophilicity, and so on [22,23,24,25,26,27]. Thus, a tremendous amount of fluorination reactions, including the asymmetric versions have been investigated [28,29,30]. Reflecting that alkene is a useful feedstock in organic chemistry and easy to prepare, asymmetric fluoro-functionalizations of alkenes have attracted increasing attention [31,32,33,34,35,36,37]. However, there is no report of the asymmetric fluorocyclization of ene-oximes, while its racemic version was recently reported in 2017 [38]. 

**Scheme 1:**
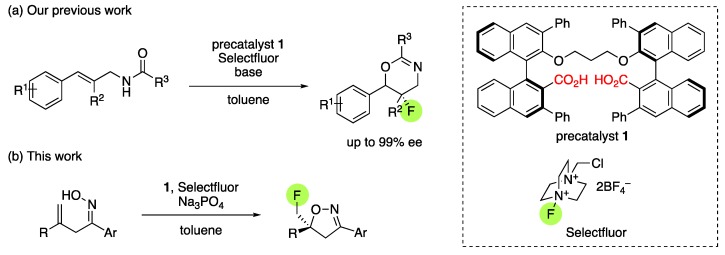
Fluorofunctionalizations of alkenes with linked-binaphthyl dicarboxylic acid 1 and Selectfluor: (**a**) Asymmetric 6-*endo*-type fluorocyclization of allylic amides; (**b**) Asymmetric 5-*exo*-fluorocyclization of ene-oximes.

Our recent research interests include the asymmetric fluorofunctionalization of alkenes by phase-transfer catalysis. In 2015, we reported the first successful example of asymmetric fluorolactonization of ene-carboxylic acids using a hydroxymethyl carboxylate phase-transfer catalyst [39]. Based on this study, we recently developed a linked-binaphthyl dicarboxylic acid precatalyst **1**, which was proven to be highly effective for the asymmetric fluorocyclization and the deprotonative fluorination of allylic amides (Scheme 1a) [40,41]. In these reactions, hydrogen bonding between the catalyst and the substrate was considered to be crucial for high asymmetric induction. Considering p*K*a values of amide and oxime, we anticipated that oxime could interact with our anionic phase-transfer catalyst through hydrogen bonds, which would define the conformation of the substrate and/or a fluorinated carbocation intermediate. Our previous study suggested that the fluorocyclization of alkenes proceeds via the formation of a fluoro-carbocation intermediate [40,41]. If this is the case, hydrogen bond interaction of the cationic intermediate with the catalyst seems essential, because the intramolecular cyclization step is an enantio-determining step. Herein, we report our effort to develop the enantioselective 5-*exo*-fluorocyclization of ene-oximes to provide fluorinated isoxazolines (Scheme 1b).

## 2. Results and Discussion

Ene-oxime **2a** [21] was chosen as a test substrate to optimize the reaction conditions (Table 1). At first, the reaction was carried out with 1 under the previous cyclization conditions [40] and the desired product, **3a**, was obtained with 58% ee (entry 1). In this reaction, undesired byproducts were simultaneously observed by ^1^H NMR analysis of the crude mixture. Although these byproducts could not be purified at this point, ^1^H and ^19^F NMR analyses suggested that the byproducts were deprotonative fluorination products **4a**–**6a**. The enantioselectivity observed in chlorobenzene and benzene was almost the same with that in toluene, but the reaction rate became somewhat slower (entries 1–3). Use of CH_2_Cl_2_ and THF resulted in low chemical yield (entries 4 and 5). While Na_2_SO_4_ had a positive effect in improving the chemical yield in our previous case [40,41], it did not affect the reaction efficiency in the present reaction (entries 1 and 6). Among bases tested, Na_3_PO_4_ was found to be the base of choice in terms of the chemical yield of the desired product **3a** (entries 6–11). The enantioselectivity was almost similar (59%–61%), irrespective of the basicity and counter cation. Proton sponge provided a low yield and a low enantioselectivity under the described conditions (entry 11). As seen in entry 12, the reaction rate became slower at 15 °C, but a better enantioselectivity was observed (69% ee). In all cases, the yields based on the recovered starting material were around 65%. Interestingly, the ee of **6a** was as high as 77% under the optimized conditions (entry 12), albeit in only 4% yield. It should be noted that phosphoric acid **7**, which is a commonly used precursor of anionic phase transfer catalysts [33], did not promote the present reaction (entry 14), confirming the better performance of our dicarboxylate catalyst.

**Scheme 2:**
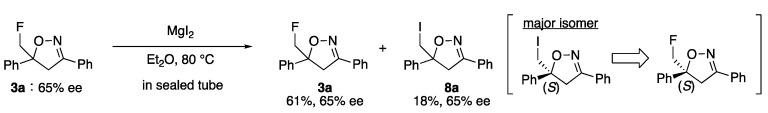
Conversion of 3a to 8a to determine the stereochemistry of the major product.

To determine the absolute stereochemistry of the major isomer, the fluorinated isoxazoline **3a** was transformed to the corresponding iodinated isoxazoline **8a** with MgI_2_ using a sealed tube at 80 °C [42] (Scheme 2). Although the conversion was modest, **8a** could be obtained without erosion of the ee value. The stereochemistry of the major isomer was determined to be *S* by comparing the retention time of HPLC analysis with that reported in the literature [21]. This result indicates that the major isomer of the present fluorocyclization is *S*. 

**Figure 1:**
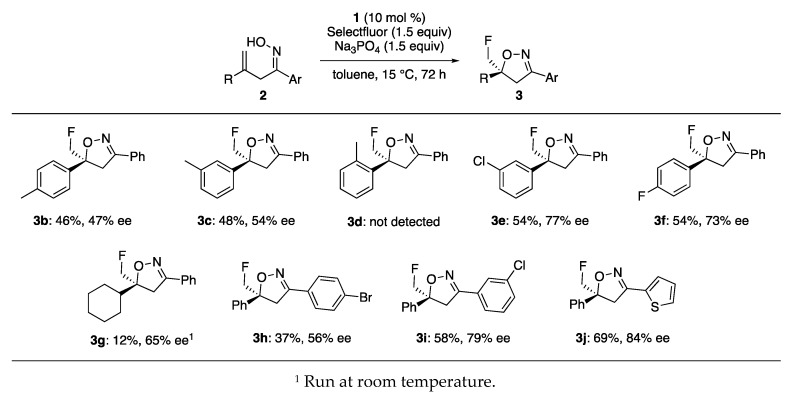
Fluorocyclization of ene-oximes.

Having optimized the reaction conditions, other ene-oximes were transformed to the corresponding fluorinated isoxazolines (Figure 1). Reactions of *para*- and *meta*-methylated substrates proceeded to give the corresponding isoxazoline products with good ee values (**3b**, **3c**). However, *ortho*-substituent retarded the reaction completely, probably due to the steric repulsion (**3d**). *meta*-Chlorinated and *para*-fluorinated isoxazolines were formed with 77% ee and 73% ee, respectively (**3e** and **3f**). An ene-oxime bearing a cyclohexyl group was less reactive for the fluorocyclization and **3g** was obtained in only 12% yield, even at room temperature. The chemical structure on the oxime side did not have a significant impact on the enantioselectivity (**3h** and **3i**). To our delight, a substrate having a thiophen group provided **3j** in 69% with 84% ee. We additionally performed the SDE (self-disproportionation of enantiomer) test by achiral column chromatography [43]. The difference between the first and the last fractions was less than 1% ee, suggesting that SDE did not occur. As usual, all fractions were collected after the chromatographic purification. 

**Scheme 3:**
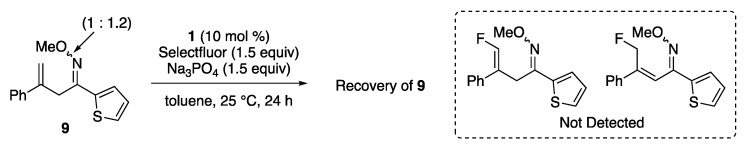
Reaction of *O*-methylated compound.

To confirm the importance of hydrogen bond interaction between the oxime and the dianionic catalyst **1**, the following control experiment was carried out. Thus, when *O*-methylated compound **9** was subjected to the described reaction conditions, no reaction occurred at all and the starting material **9** was just recovered (Scheme 3). Since even deprotonated products were not formed, it is likely that the hydrogen bond interaction has an important role in accelerating the fluorination step, in addition to the enantioselectivity control.

## 3. Materials and Methods

### 3.1. General Information

^1^H and ^19^F NMR spectra were measured on a JEOL ECX-500 spectrometer at 500 and 470 MHz, respectively. ^13^C NMR spectra were recorded on a JEOL JNM-ECX-500 spectrometer at 125 MHz. Chemical shifts were reported in parts per million (ppm) downfield from TMS (*δ* = 0) for ^1^H NMR. For ^13^C NMR, chemical shifts were reported in the scale relative to CDCl_3_. For ^19^F NMR, chemical shifts were reported in a scale relative to CFCl_3_ external standard (*δ* = 0 ppm). Column chromatography was performed with silica gel N-60 (40–100 μm) purchased from Kanto Chemical Co., Inc. TLC analysis was performed on Silica gel 60 F_254_-coated glass plates (Merck). Visualization was accomplished by means of ultraviolet (UV) irradiation at 254 nm or by spraying an ethanol solution of 12-molybdo(VI)phosphoric acid as a developing agent.

Dehydrated dichloromethane (CH_2_Cl_2_) and toluene were purchased from Wako Pure Chemical Industries, Ltd. Dehydrated tetrahydrofuran (THF), diethyl ether (Et_2_O), and benzene were purchased from Kanto Chemical Co., Inc. Selectfluor was purchased from Aldrich. Other reagents were purified by usual methods.

The substrates were synthesized according to the literature [21]. The catalysts were synthesized according to the literature [40]. The ^1^H, ^13^C and ^19^F NMR spectra and HPLC data of compounds **3** are available in the Supplementary Material.

### 3.2. Asymmetric Fluorocyclization of Ene-Oximes

To a solution of **2a** (23.7 mg, 0.1 mmol), **1** (9.7 mg, 10 mol %), and Na_3_PO_4_ (24.6 mg, 1.5 equiv) in toluene (1 mL), was added Selectfluor (53.1 mg, 1.5 equiv) at 15 °C under Ar atmosphere. After stirring for 72 h at 15 °C, the reaction mixture was diluted with EtOAc and filtrated through a pad of Celite. The filtrate was concentrated under reduced pressure. The resulting residue was purified by column chromatography on silica gel (*n*-hexane/ethyl acetate = 20/1 → 15/1 → 10/1) to provide **3a** as a colorless solid (14.8 mg, 58%). The ee value was determined by chiral HPLC analysis.

5-(Fluoromethyl)-3,5-diphenylisoxazoline (**3a**). Colorless solid (58%, 14.8 mg). [*α*]^27^_D_ = 29.7 (c = 0.47, CHCl_3_). The ee value (69% ee) was determined by HPLC analysis using a chiralpack IG-3 (*n*-hexane/*i*-PrOH = 80:20, flow rate = 1.0 mL/min, t(minor) = 11.4 min, t(major) = 20.6 min). ^1^H NMR (500 MHz, CDCl_3_) *δ* = 7.69–7.67 (m, 2H), 7.53–7.51 (m, 2H), 7.43–7.38 (m, 5H), 7.36–7.33 (m, 1H), 4.64 (dd, *J* = 9.7, 47.5 Hz, 1H), 4.60 (dd, *J* = 9.7, 47.5 Hz, 1H), 3.89 (d, *J* = 16.6 Hz, 1H), 3.53 (dd, *J* = 2.3, 16.6 Hz, 1H). ^13^C NMR (125 MHz, CDCl_3_) *δ* = 156.2, 140.1 (d, *J* = 3.6 Hz), 130.3, 129.3, 128.7, 128.7, 128.4, 126.7, 125.5, 88.9 (d, *J* = 18.0 Hz), 85.5 (d, *J* = 183.5 Hz), 42.9 (d, *J* = 3.6 Hz). ^19^F NMR (470 MHz, CDCl_3_) *δ* = −221.3 (t, *J* = 47.5 Hz, 1F). IR (neat): 2941, 1599, 1447, 1362, 1020, 980, 912 cm^−1^. HRMS (ESI) m/z calcd. for C_16_H_15_FNO [M + H^+^]: 256.1132, found: 256.1131.

5-(Fluoromethyl)-3-phenyl-5-(p-tolyl)isoxazoline (**3b**). Colorless solid (46%, 12.7 mg). [*α*]^29^_D_ = 31.8 (c = 0.38, CHCl_3_). The ee value (47% ee) was determined by HPLC analysis using a chiralpack IG-3 (*n*-hexane/*i*-PrOH = 80:20, flow rate = 1.0 mL/min, t(minor) = 13.4 min, t(major) = 23.5 min). ^1^H NMR (500 MHz, CDCl_3_) *δ* = 7.67–7.66 (m, 2H), 7.42–7.37 (m, 5H), 7.21 (d, *J* = 8.0 Hz, 2H), 4.61 (dd, *J* = 10.0, 47.4 Hz, 1H), 4.57 (dd, *J* = 10.0, 47.4 Hz, 1H) 3.86 (d, *J* = 16.6 Hz, 1H), 3.51 (dd, *J* = 1.7, 16.6 Hz, 1H), 2.35 (s, 3H). ^13^C NMR (125 MHz, CDCl_3_) *δ* = 156.2, 138.2, 137.1 (d, *J* = 3.6 Hz), 130.2, 129.4, 129.3, 128.7, 126.6, 125.4, 88.9 (d, *J* = 18.0 Hz), 85.5 (d, *J* = 183.5 Hz), 42.8 (d, *J* = 3.6 Hz), 21.0. ^19^F NMR (470 MHz, CDCl_3_) *δ* = −220.9 (t, *J* = 47.4 Hz, 1F). IR (neat): 2922, 1570, 1514, 1447, 1358, 978, 906 cm^−1^. HRMS (ESI) m/z calcd. for C_17_H_17_FNO [M + H^+^]: 270.1289, found: 270.1287.

5-(Fluoromethyl)-3-phenyl-5-(m-tolyl)isoxazoline (**3c**). Colorless solid (48%, 12.9 mg). [*α*]^28^_D_ = 21.4 (c = 0.50, CHCl_3_). The ee value (54% ee) was determined by HPLC analysis using a chiralpack IG-3 (*n*-hexane/*i*-PrOH = 80:20, flow rate = 1.0 mL/min, t(minor) = 8.7 min, t(major) = 12.4 min). ^1^H NMR (500 MHz, CDCl_3_) *δ* = 7.69–7.68 (m, 2H), 7.41–7.39 (m, 3H), 7.35 (s, 1H), 7.30–7.29 (m, 2H), 7.17-7.15 (m, 1H), 4.63 (dd, *J* = 10.0, 47.6 Hz, 1H), 4.60 (dd, *J* = 10.0, 47.6 Hz, 1H), 3.88 (d, *J* = 16.6 Hz, 1H), 3.53 (dd, *J* = 2.3, 16.6 Hz, 1H), 2.39 (s, 3H). ^13^C NMR (125 MHz, CDCl_3_) *δ* = 156.2, 140.0 (d, *J* = 3.6 Hz), 138.5, 130.2, 129.3, 129.1, 128.7, 128.6, 126.6, 126.1, 122.5, 88.9 (d, *J* = 18.0 Hz), 85.6 (d, *J* = 183.5 Hz), 42.8 (d, *J* = 3.6 Hz), 21.5. ^19^F NMR (470 MHz, CDCl_3_) *δ* = −221.1 (t, *J* = 47.6 Hz, 1F). IR (neat): 2955, 1600, 1487, 1447, 1364, 1011, 922 cm^−1^. HRMS (ESI) m/z calcd. for C_17_H_17_FNO [M + H^+^]: 270.1289, found: 270.1286.

5-(3-Chlorophenyl)-5-(fluoromethyl)-3-phenylisoxazoline (**3e**). Colorless oil (54%, 15.6 mg). [*α*]^27^_D_ = 33.3 (c = 0.19, CHCl_3_). The ee value (77% ee) was determined by HPLC analysis using a chiralpack IG-3 (*n*-hexane/*i*-PrOH = 80:20, flow rate = 1.0 mL/min, t(minor) = 8.8 min, t(major) = 10.2 min). ^1^H NMR (500 MHz, CDCl_3_) *δ* = 7.68–7.66 (m, 2H), 7.53 (s, 1H), 7.41–7.39 (m, 4H), 7.36–7.31 (m, 2H), 4.62 (dd, *J* = 10.0, 47.0 Hz, 1H), 4.56 (dd, *J* = 10.0, 47.0 Hz, 1H), 3.88 (d, *J* = 16.6 Hz, 1H), 3.50 (dd, *J* = 2.3, 16.6 Hz, 1H). ^13^C NMR (125 MHz, CDCl_3_) *δ* = 156.2, 142.3 (d, *J* = 2.4 Hz), 134.8, 130.4, 130.1, 128.9, 128.8, 128.6, 126.7, 125.9, 123.7, 88.3 (d, *J* = 19.2 Hz), 85.1 (d, *J* = 183.5 Hz), 43.1 (d, *J* = 3.6 Hz). ^19^F NMR (470 MHz, CDCl_3_) *δ* = −221.6 (t, *J* = 47.0 Hz, 1F). IR (neat): 2940, 1597, 1572, 1447, 1358, 1034, 995, 910 cm^−1^. HRMS (ESI) m/z calcd. for C_16_H_14_ClFNO [M + H^+^]: 290.0742, found: 290.0743.

5-(Fluoromethyl)-5-(4-fluorophenyl)-3-phenylisoxazoline (**3f**). Colorless solid (54%, 14.8 mg). [*α*]^28^_D_ = 27.6 (c = 0.40, CHCl_3_). The ee value (73% ee) was determined by HPLC analysis using a chiralpack IG-3 (*n*-hexane/*i*-PrOH = 80:20, flow rate = 1.0 mL/min, t(minor) = 10.1 min, t(major) = 17.1 min). ^1^H NMR (500 MHz, CDCl_3_) *δ* = 7.68–7.66 (m, 2H), 7.51–7.48 (m, 2H), 7.42–7.38 (m, 3H), 7.11–7.08 (m, 2H), 4.61 (dd, *J* = 9.7, 47.5 Hz, 1H), 4.57 (dd, *J* = 9.7, 47.5 Hz, 1H), 3.88 (d, *J* = 16.6 Hz, 1H), 3.49 (dd, *J* = 2.3, 16.6 Hz, 1H). ^13^C NMR (125 MHz, CDCl_3_) *δ* = 162.6 (d, *J* = 247.1 Hz), 156.2, 136.0 (t, *J* = 3.6 Hz), 130.4, 129.1, 128.8, 127.4 (d, *J* = 8.4 Hz), 126.7, 115.7 (d, *J* = 21.6 Hz), 88.4 (d, *J* = 19.2 Hz), 85.3 (d, *J* = 183.5 Hz), 43.1 (d, *J* = 3.6 Hz). ^19^F NMR (470 MHz, CDCl_3_) *δ* = −113.5–−113.6 (m, 1F), −221.1 (t, *J* = 47.5 Hz, 1F). IR (neat): 1601, 1508, 1447, 1360, 1221, 1159, 1028, 918 cm^−1^. HRMS (ESI) m/z calcd. for C_16_H_14_F_2_NO [M + H^+^]: 274.1038, found: 274.1034.

5-Cyclohexyl-5-(fluoromethyl)-3-phenylisoxazoline (**3g**). Colorless oil (12%, 3.1 mg). [*α*]^29^_D_ = 45.8 (c = 0.34, CHCl_3_). The ee value (65% ee) was determined by HPLC analysis using a chiralpack IG-3 (*n*-hexane/*i*-PrOH = 80:20, flow rate = 1.0 mL/min, t(minor) = 8.2 min, t(minor) = 9.2 min). ^1^H NMR (500 MHz, CDCl_3_) *δ* = 7.68–7.66 (m, 2H), 7.41–7.39 (m, 3H), 4.53 (dd, *J* = 9.7, 47.0 Hz, 1H), 4.49 (dd, *J* = 9.7, 47.0 Hz, 1H), 3.22 (d, *J* = 1.7 Hz, 2H), 1.82–1.78 (m, 5H), 1.71–1.69 (m, 1H), 1.32–1.05 (m, 5H). ^13^C NMR (125 MHz, CDCl_3_) *δ* = 155.7, 130.0, 129.6, 128.7, 126.5, 90.2 (d, *J* = 16.8 Hz), 84.2 (d, *J* = 177.5 Hz), 42.6 (d, *J* = 2.4 Hz), 37.9 (d, *J* = 6.0 Hz), 27.2, 26.5, 26.2, 26.1, 26.1. ^19^F NMR (470 MHz, CDCl_3_) *δ* = −229.6 (t, *J* = 47.0 Hz, 1F). IR (neat): 2922, 1595, 1447, 1360, 1009, 926 cm^−1^. HRMS (ESI) m/z calcd. for C_16_H_21_FNO [M + H^+^]: 262.1602, found: 262.1609.

3-(4-Bromophenyl)-5-(fluoromethyl)-5-phenylisoxazoline (**3h**). Colorless solid (37%, 12.4 mg). [*α*]^28^_D_ = 22.5 (c = 0.39, CHCl_3_). The ee value (56% ee) was determined by HPLC analysis using a chiralpack IG-3 (*n*-hexane/*i*-PrOH = 80:20, flow rate = 1.0 mL/min, t(minor) = 12.9 min, t(major) = 20.4 min). ^1^H NMR (500 MHz, CDCl_3_) *δ* = 7.55–7.49 (m, 6H), 7.43–7.40 (m, 2H), 7.36–7.33 (m, 1H), 4.63 (dd, *J* = 10.3, 47.1 Hz, 1H), 4.59 (dd, *J* = 10.3, 47.1 Hz, 1H), 3.86 (d, *J* = 16.6 Hz, 1H), 3.50 (dd, *J* = 2.3, 16.6 Hz, 1H). ^13^C NMR (125 MHz, CDCl_3_) *δ* = 155.3, 139.8 (d, *J* = 4.8 Hz), 131.9, 128.8, 128.5, 128.2, 128.1, 125.4, 124.5, 89.3 (d, *J* = 18.0 Hz), 85.5 (d, *J* = 184.7 Hz), 42.6 (d, *J* = 3.6 Hz). ^19^F NMR (470 MHz, CDCl_3_) *δ* = −221.2 (t, *J* = 47.1 Hz, 1F). IR (neat): 2951, 1589, 1489, 1447, 1396, 1356, 1246, 989 cm^−1^. HRMS (ESI) m/z calcd. for C_16_H_14_BrFNO [M + H^+^]: 334.0237, found: 334.0237.

3-(3-Chlorophenyl)-5-(fluoromethyl)-5-phenylisoxazoline (**3i**). Colorless oil (58%, 16.8 mg). [*α*]^28^_D_ = 43.5 (c = 0.30, CHCl_3_). The ee value (79% ee) was determined by HPLC analysis using a chiralpack IG-3 (*n*-hexane/*i*-PrOH = 80:20, flow rate = 1.0 mL/min, t(minor) = 8.1 min, t(major) =9.7 min). ^1^H NMR (500 MHz, CDCl_3_) *δ* = 7.66 (s, 1H), 7.57 (d, *J* = 7.5 Hz, 1H), 7.50 (d, *J* = 8.0 Hz, 2H), 7.43–7.40 (m, 2H), 7.38–7.31 (m, 3H), 4.64 (dd, *J* = 10.3, 47.1 Hz, 1H), 4.60 (dd, *J* = 10.3, 47.1 Hz, 1H), 3.87 (d, *J* = 16.6 Hz, 1H), 3.51 (dd, *J* = 2.3, 16.6 Hz, 1H). ^13^C NMR (125 MHz, CDCl_3_) *δ* = 155.2, 139.7 (d, *J* = 3.6 Hz), 134.7, 131.0, 130.2, 130.0, 128.8, 128.5, 126.7, 125.4, 124.7, 89.4 (d, *J* = 18.0 Hz), 85.5 (d, *J* = 184.7 Hz), 42.5 (d, *J* = 3.6 Hz). ^19^F NMR (470 MHz, CDCl_3_) *δ* = −221.2 (t, *J* = 47.1 Hz). IR (neat): 3063, 2947, 1595, 1560, 1429, 1344, 1028, 918 cm^−1^. HRMS (ESI) m/z calcd. for C_16_H_14_ClFNO [M + H^+^]: 290.0742, found: 290.0724.

5-(fluoromethyl)-5-phenyl-3-(3-thiophen-2-yl)isoxazoline (**3j**). Colorless solid (69%, 18.0 mg). [*α*]^28^_D_ = 14.3 (c = 0.25, CHCl_3_). The ee value (84% ee) was determined by HPLC analysis using a chiralpack IG-3 (*n*-hexane/*i*-PrOH = 80:20, flow rate = 1.0 mL/min, t(minor) = 11.8 min, t(major) = 21.6 min). ^1^H NMR (500 MHz, CDCl_3_) *δ* = 7.52–7.50 (m, 2H), 7.43–7.38 (m, 3H), 7.36–7.33 (m, 1H), 7.21–7.20 (m, 1H), 7.06–7.04 (m, 1H), 4.63 (dd, *J* = 10.3, 47.5 Hz, 1H), 4.59 (dd, *J* = 10.3, 47.5 Hz, 1H), 3.90 (d, *J* = 16.2 Hz, 1H), 3.53 (dd, *J* = 2.6, 16.2 Hz, 1H). ^13^C NMR (125 MHz, CDCl_3_) *δ* = 152.0, 139.8 (d, *J* = 3.6 Hz), 131.7, 128.8, 128.5, 128.5, 128.4, 127.3, 125.5, 89.1 (d, *J* = 18.1 Hz), 85.3 (d, *J* = 183.5 Hz), 43.6 (d, *J* = 3.6 Hz). ^19^F NMR (470 MHz, CDCl_3_) *δ* = −221.1 (t, *J* = 47.5 Hz). IR (neat): 3084, 2943, 1600, 1491, 1437, 1024, 982, 907 cm^−1^. HRMS (ESI) m/z calcd. for C_14_H_13_FNOS [M + H^+^]: 262.0696, found: 262.0695.

## 4. Conclusions

In this paper, we have demonstrated the enantioselective 5-*exo*-fluorocyclization of ene-oximes using the linked-binaphthyl dicarboxylic acid precatalyst **1**. The corresponding fluorinated isoxazolines were obtained with up to 84% ee, and the stereochemistry of the major isomers were determined to be *S* after transformation to known isoxazoline **8a** with an iodomethyl unit. A control experiment revealed that hydrogen bond interaction of the oxime group is extremely important for the reaction acceleration and the enantioselectivity control. Further applications of the present fluorinating system are underway in our laboratory.

## Figures and Tables

**Table 1:**
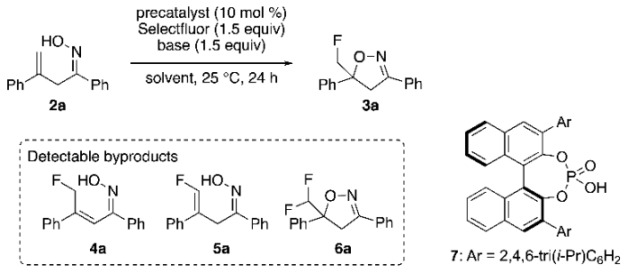
Optimization of the reaction conditions. ^1^

Entry	Precatalyst	Solvent	Base	Yield (%) ^2^	Ee (%) ^3^
1 ^4^	**1**	toluene	Na_3_PO_4_	39	58
2 ^4^	**1**	chlorobenzene	Na_3_PO_4_	26	58
3 ^4^	**1**	benzene	Na_3_PO_4_	30	57
4 ^4^	**1**	CH_2_Cl_2_	Na_3_PO_4_	11	-
5 ^4^	**1**	THF	Na_3_PO_4_	3	-
6	**1**	toluene	Na_3_PO_4_	42	59
7	**1**	toluene	Na_2_HPO_4_	19	61
8	**1**	toluene	Na_2_CO_3_	35	61
9	**1**	toluene	K_3_PO_4_	24	59
10	**1**	toluene	K_2_CO_3_	3	-
11	**1**	toluene	proton sponge	17	7
12 ^5^	**1**	toluene	Na_3_PO_4_	63 (58) ^6^	69
13 ^5^	**1**	toluene	Na_2_CO_3_	62 (58) ^6^	65
14 ^5^	**7**	toluene	Na_3_PO_4_	2	-

^1^ The reactions were carried out with **2a** (0.1 mmol), precatalyst (10 mol %), Selectfluor (1.5 equiv), and base (1.5 equiv) at 25 °C, unless otherwise mentioned. ^2^ The yields were determined by ^1^H NMR analysis using 1,1,2,2-tetrabromoethane as an internal standard. ^3^ The ee values were determined by HPLC analysis using a chiral stationary column. ^4^ Run with Na_2_SO_4_. ^5^ Run at 15 °C for 72 h. ^6^ Isolated yield.

## Supplementary Materials

The following are available online, ^1^H, ^13^C, and ^19^F NMR spectra of compounds **3** and HPLC data of compounds **3**.
