# Supplementary material for: Enantioselective 5-exo-Fluorocyclization of Ene-Oximes
Source: Molecules. 2019 Sep 24;24(19):3464. doi: 10.3390/molecules24193464 (PMC6804199; doi:10.3390/molecules24193464)
Supplement: Supplementary file 1 [file molecules-24-03464-s001.pdf]

Supplementary Materials

Enantioselective 5-*exo*-Fluorocyclization of Ene-Oximes

Taiki Rouno, Tomoki Niwa, Kousuke Nishibashi, Nobuharu Yamamoto, Hiromichi Egami, and Yoshitaka Hamashima*

School of Pharmaceutical Sciences, University of Shizuoka, 52-1 Yada, Suruga-ku, Shizuoka 422-8526, Japan

**Correspondence: hamashima@u-shizuoka-ken.ac.jp*

Table of Contents

1. ^{1}H, ^{13}C, and ^{19}F NMR spectra of compounds **3**

2. HPLC data of compounds **3** and **8a**

1. ^{1}H, ^{13}C, and ^{19}F NMR spectra of compounds 3

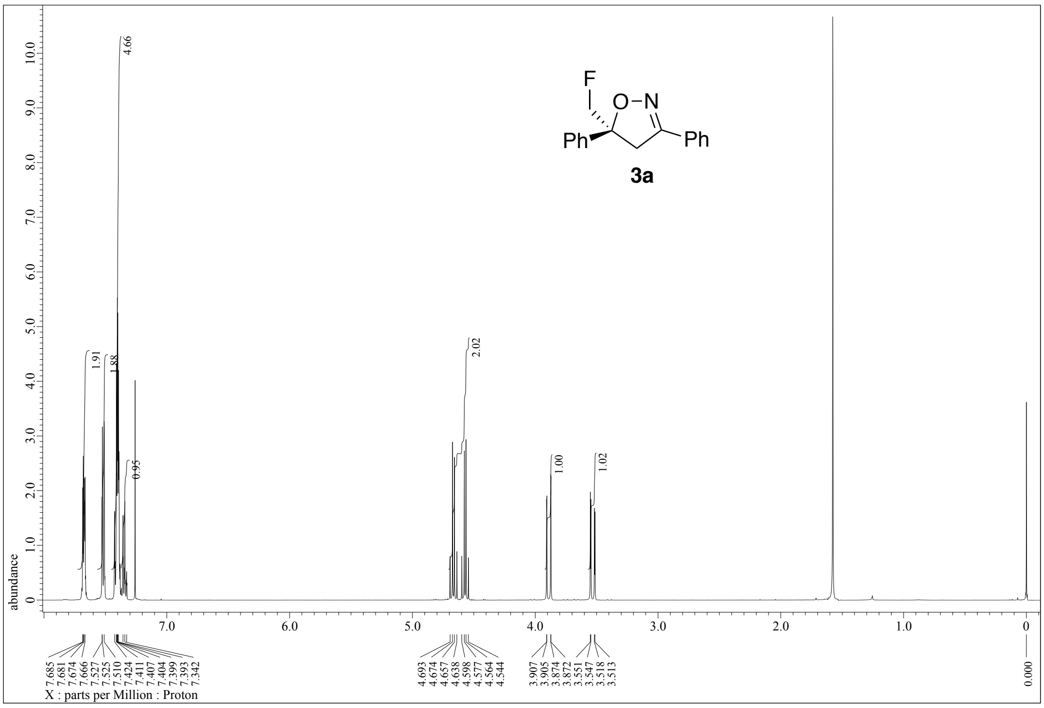

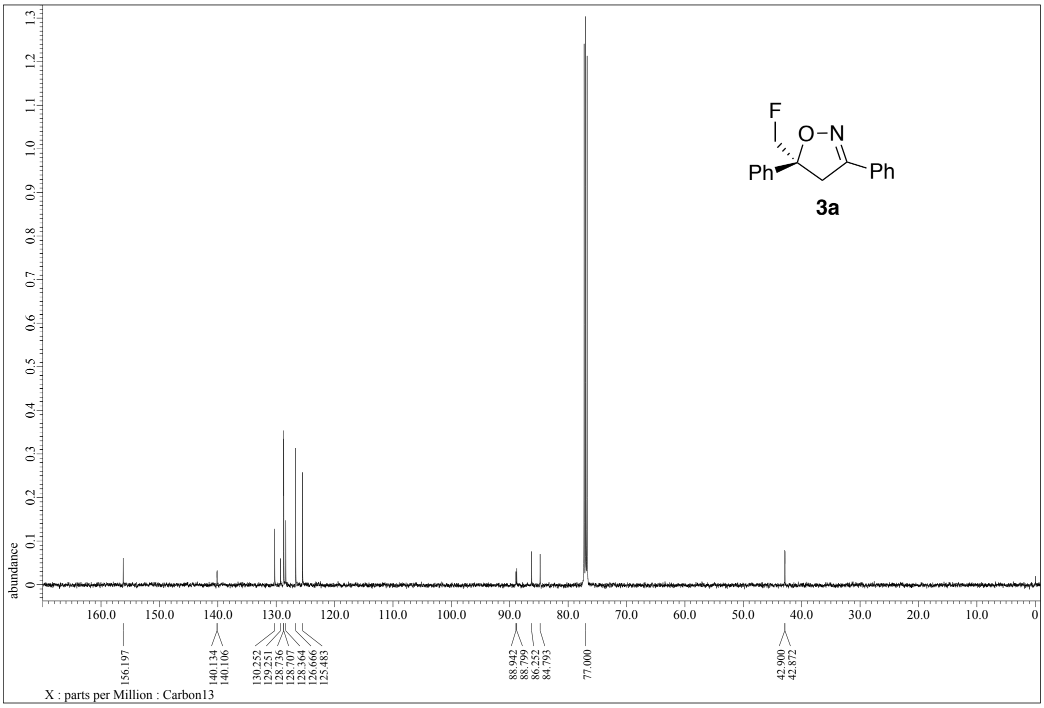

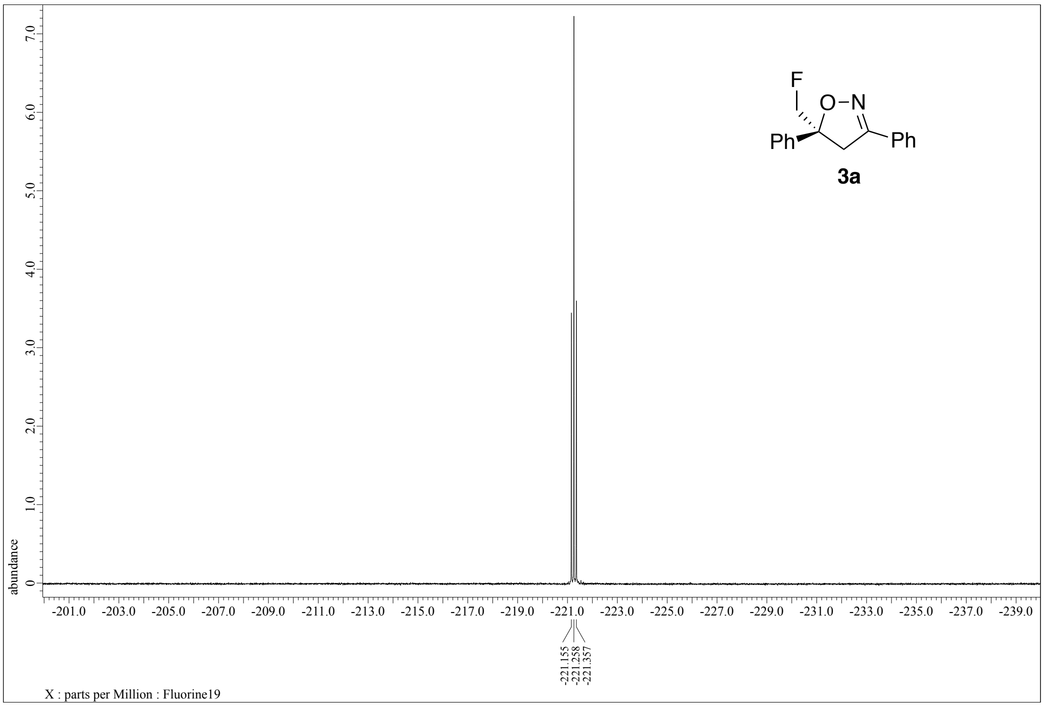
3a
Ph
O–N
F
abundance
X : parts per Million : Fluorine19
-221.155
-221.258
-221.357

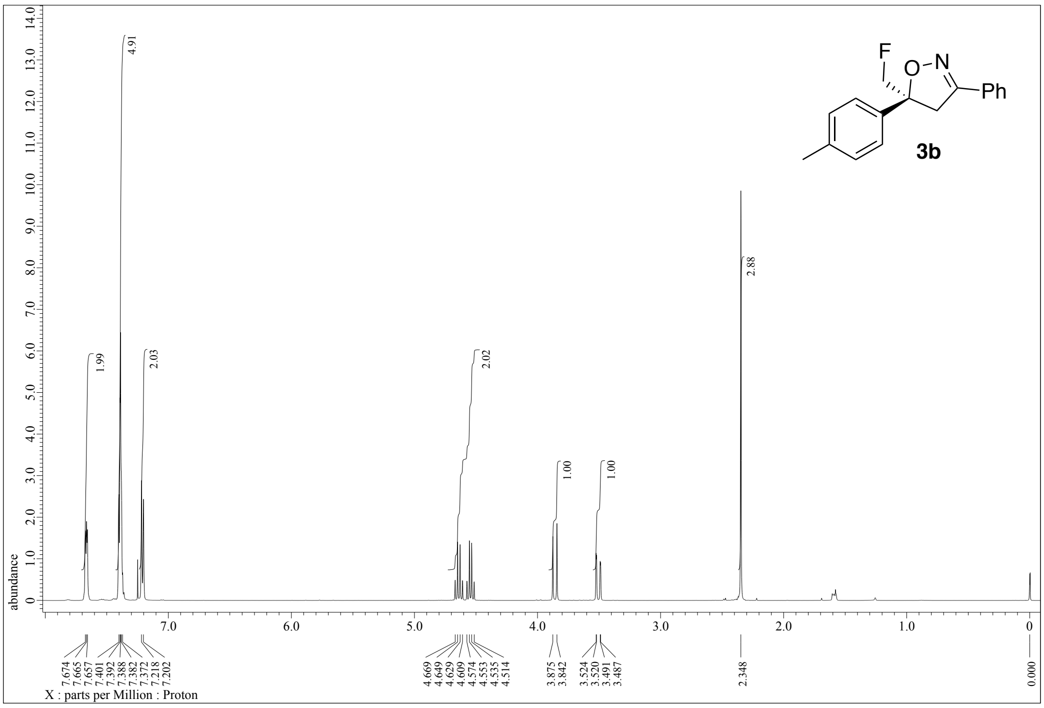
3b
Ph
O–N
F
abundance
X : parts per Million : Proton
4.91
1.99
2.03
2.02
1.00
1.00
2.88
7.674
7.665
7.657
7.401
7.392
7.388
7.382
7.372
7.218
7.202
4.669
4.649
4.629
4.609
4.574
4.553
4.535
4.514
3.875
3.842
3.524
3.520
3.491
3.487
2.348
0.000

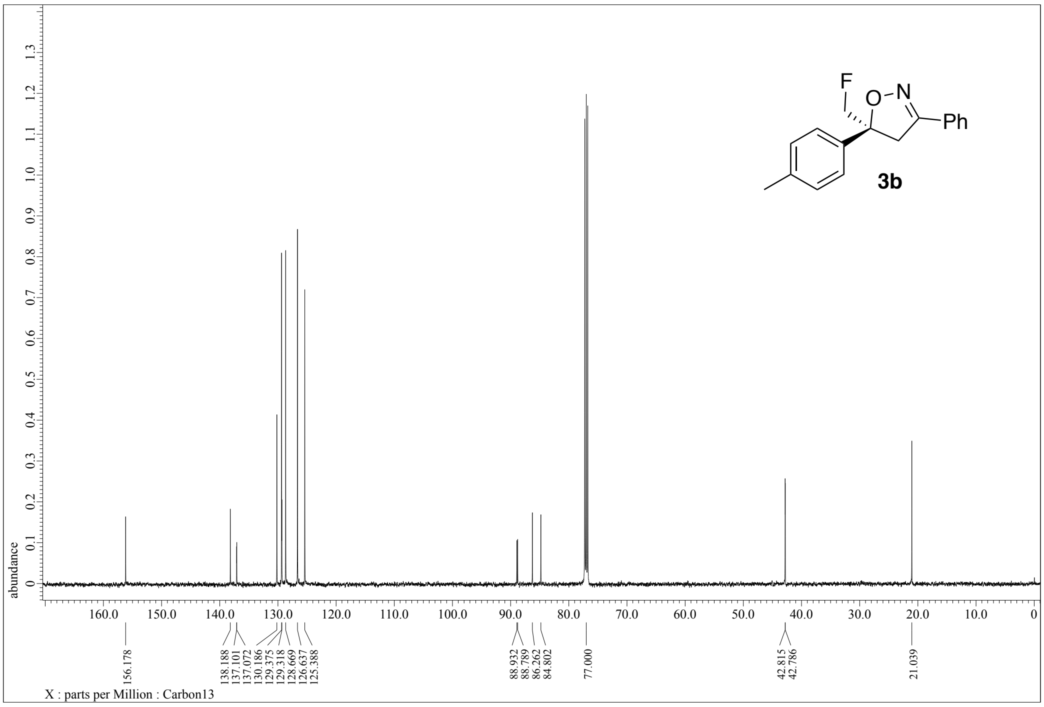
F
O
N
Ph
3b
abundance
156.178
138.188
137.101
137.072
130.186
129.375
129.318
128.669
126.637
125.388
88.932
88.789
86.262
84.802
77.000
42.815
42.786
21.039
X : parts per Million : Carbon13

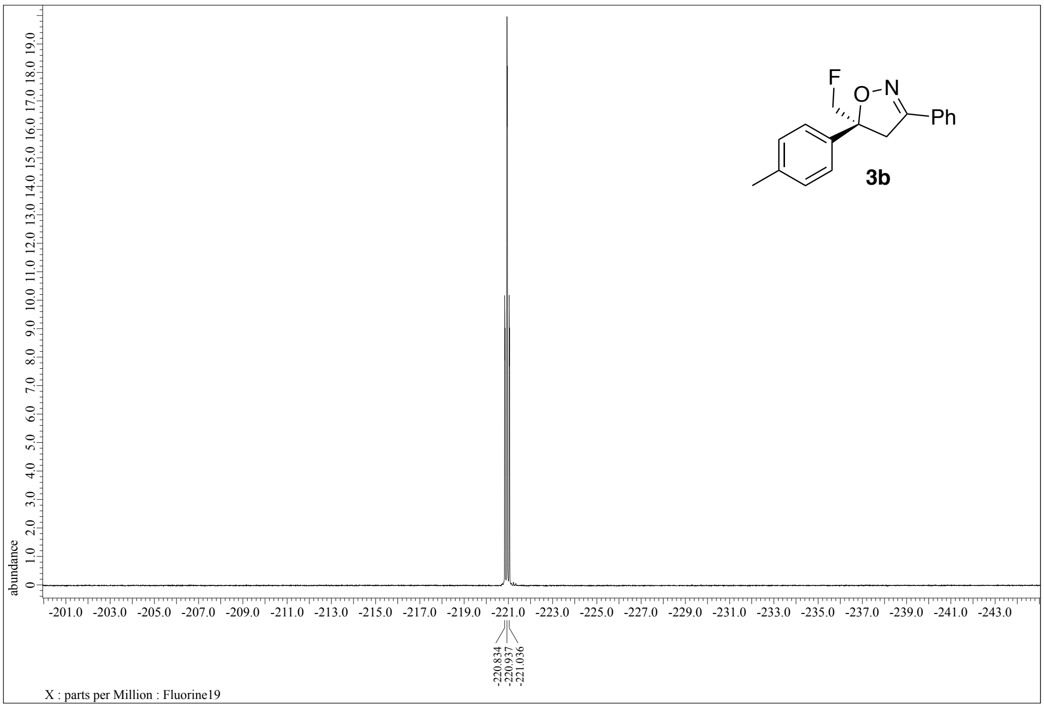
F
O
N
Ph
3b
abundance
-220.834
-220.937
-221.036
X : parts per Million : Fluorine19

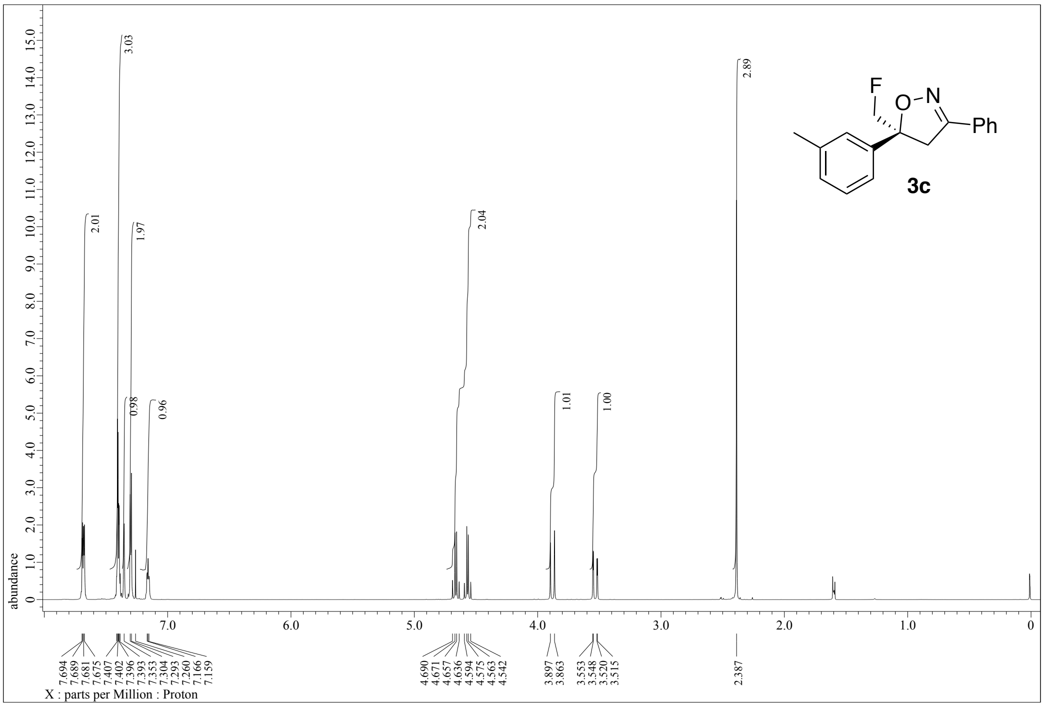

abundance
3c
Ph
X : parts per Million : Proton

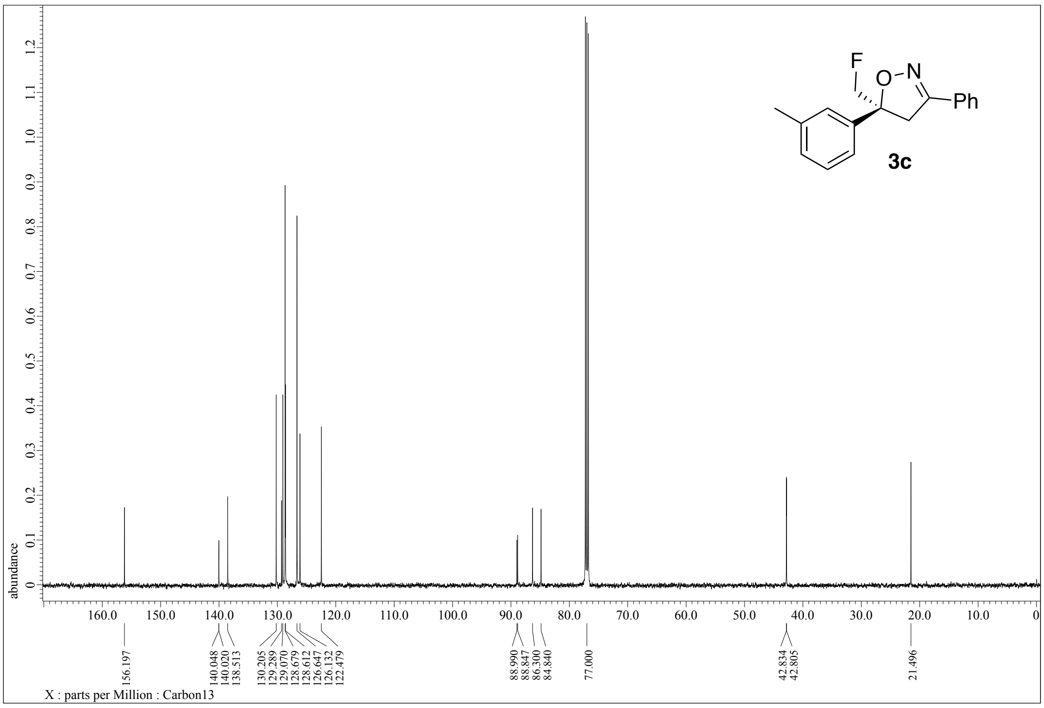

abundance
3c
Ph
X : parts per Million : Carbon13

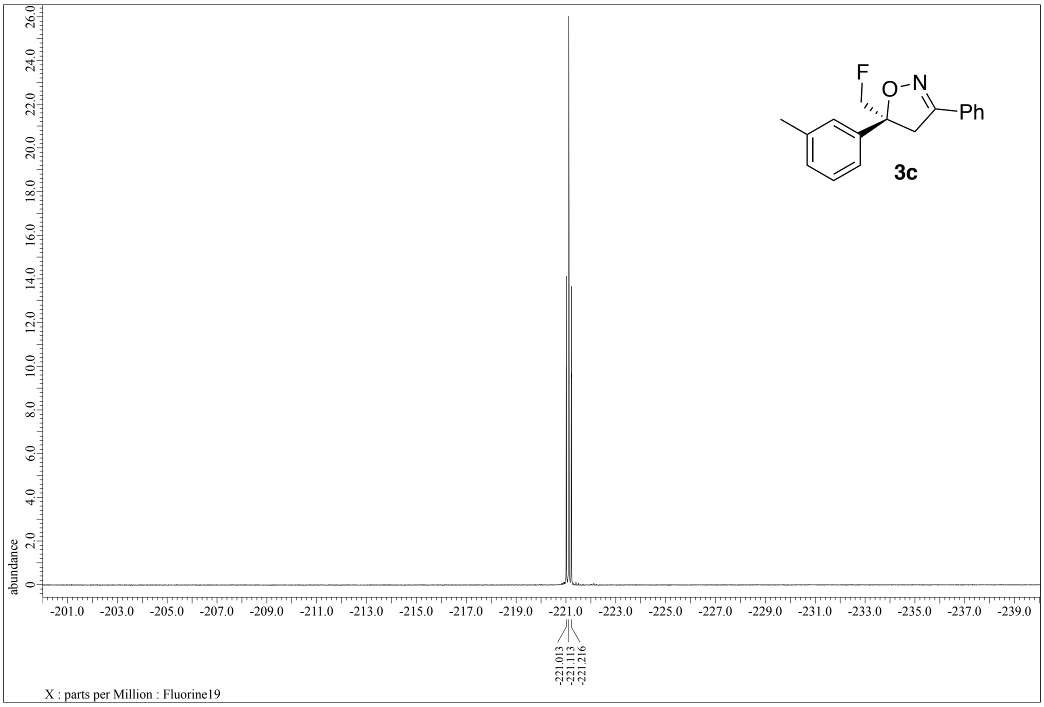

abundance
X : parts per Million : Fluorine19
-221.013
-221.113
-221.216
F
O-N
Ph
3c

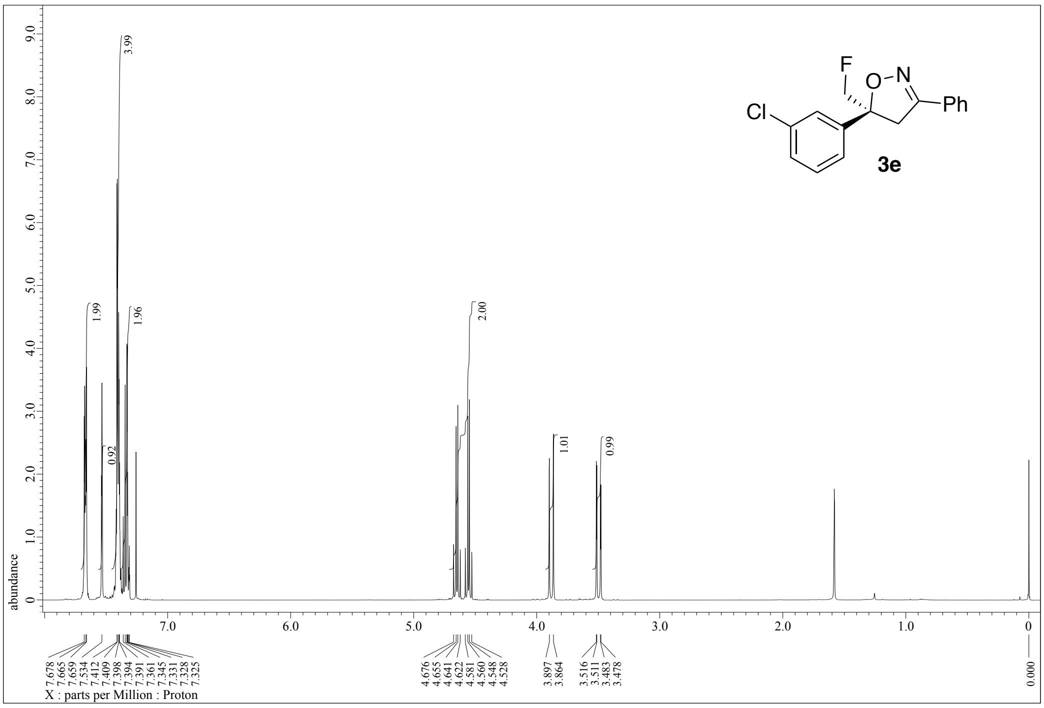

abundance
X : parts per Million : Proton
F
O-N
Ph
Cl
3e

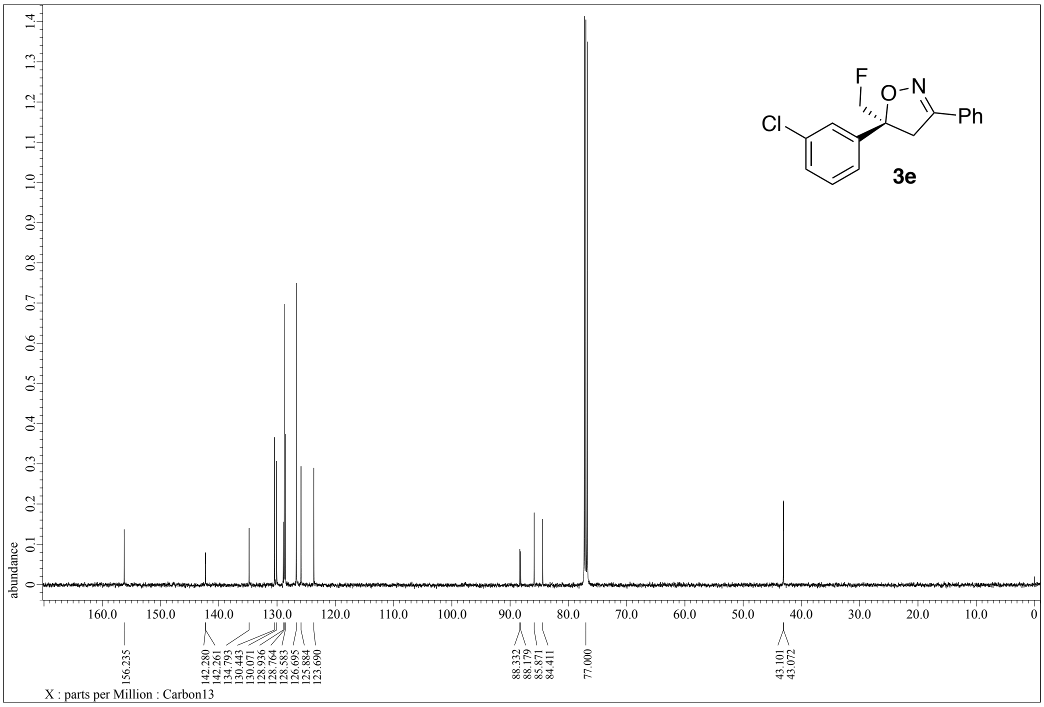
abundance
156.235
142.280
142.261
134.793
130.443
130.071
128.936
128.764
128.583
126.695
125.884
123.690
88.332
88.179
85.871
84.411
77.000
43.101
43.072
X : parts per Million : Carbon13
F
O
N
Ph
Cl
3e

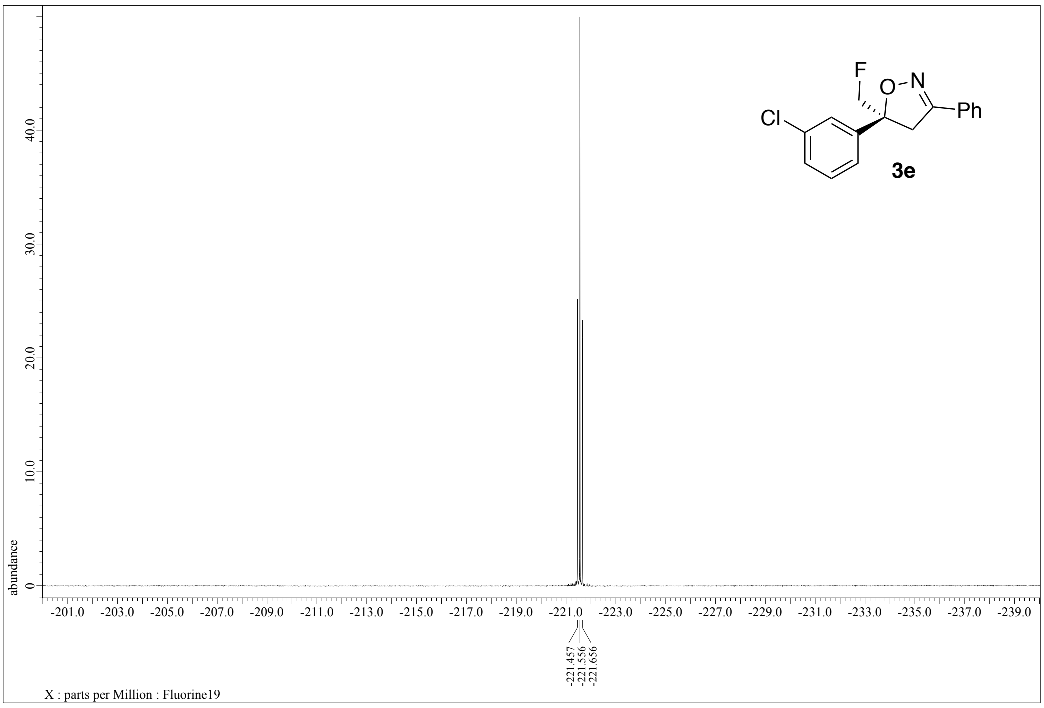
abundance
-221.457
-221.556
-221.656
X : parts per Million : Fluorine19
F
O
N
Ph
Cl
3e

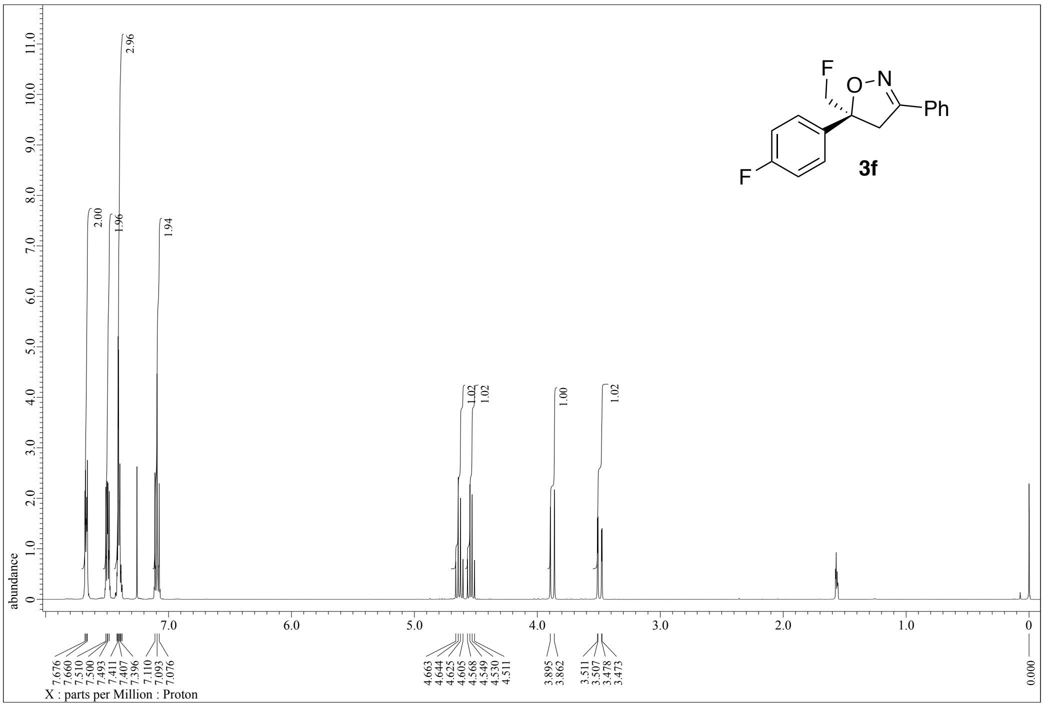
F
O–N
Ph
F
3f
abundance
X : parts per Million : Proton
2.00
1.96
2.96
1.94
1.02
1.02
1.00
1.02
7.676
7.660
7.510
7.500
7.493
7.411
7.407
7.396
7.110
7.093
7.076
4.663
4.644
4.625
4.605
4.568
4.549
4.530
4.511
3.895
3.862
3.511
3.507
3.478
3.473
0.000

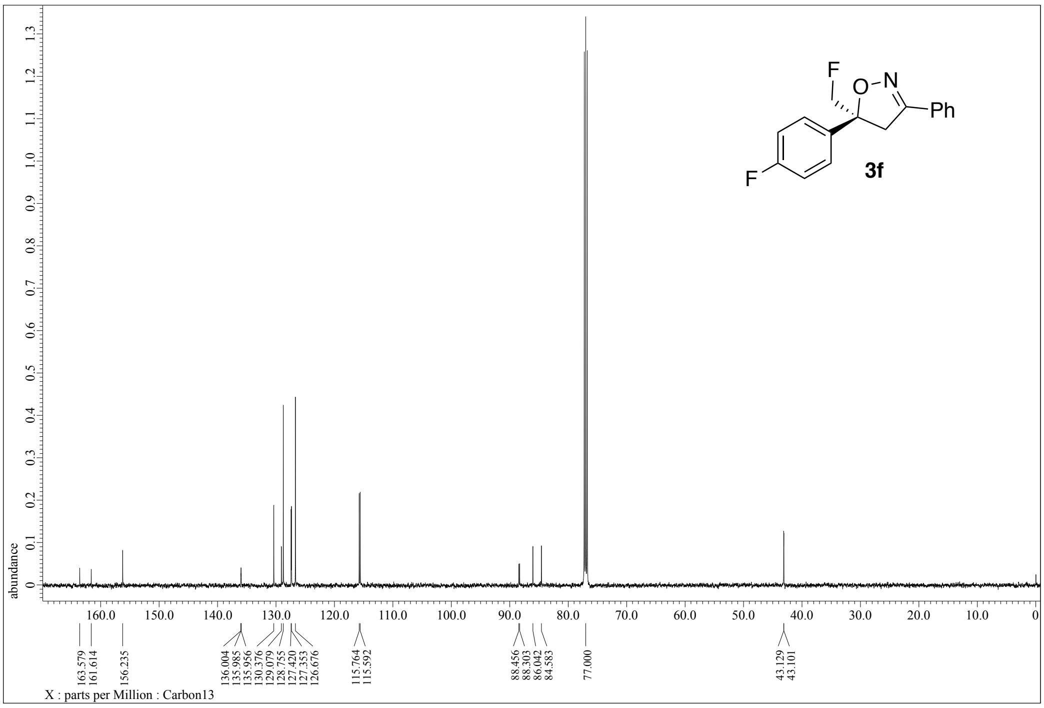
F
O–N
Ph
F
3f
abundance
X : parts per Million : Carbon13
163.579
161.614
156.235
136.004
135.985
135.956
130.376
129.079
128.755
127.420
127.353
126.676
115.764
115.592
88.456
88.303
86.042
84.583
77.000
43.129
43.101

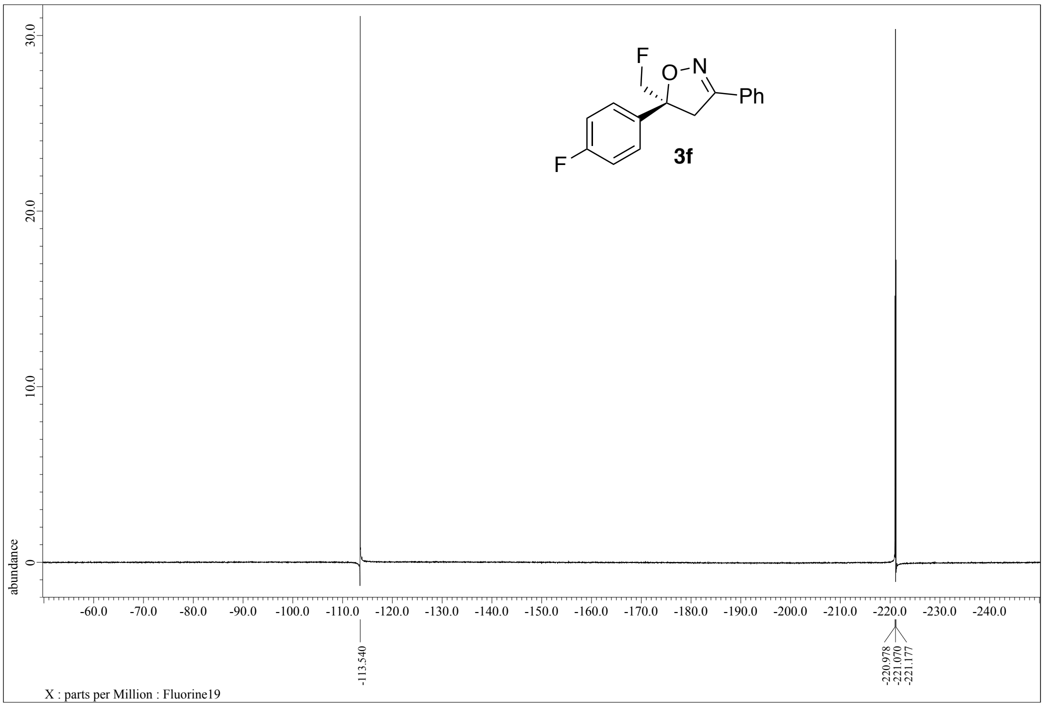
30.0
20.0
10.0
0
abundance
-60.0 -70.0 -80.0 -90.0 -100.0 -110.0 -120.0 -130.0 -140.0 -150.0 -160.0 -170.0 -180.0 -190.0 -200.0 -210.0 -220.0 -230.0 -240.0
-113.540
-220.978
-221.070
-221.177
X : parts per Million : Fluorine19
F
O-N
Ph
F
3f

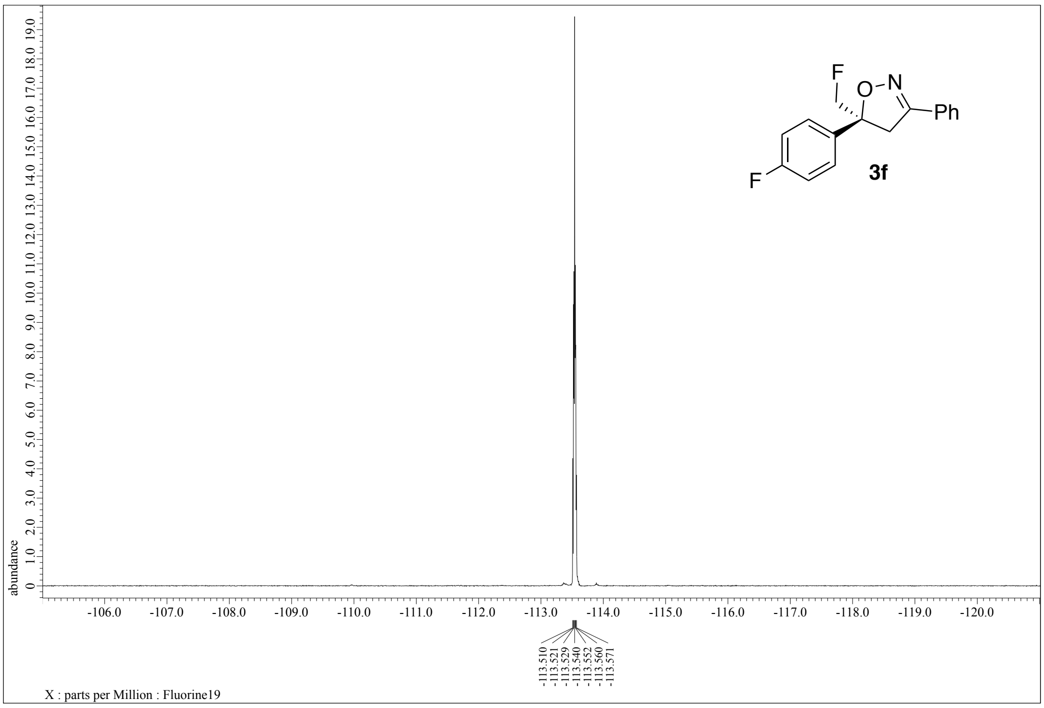
abundance
-106.0 -107.0 -108.0 -109.0 -110.0 -111.0 -112.0 -113.0 -114.0 -115.0 -116.0 -117.0 -118.0 -119.0 -120.0
-113.510
-113.521
-113.529
-113.540
-113.552
-113.560
-113.571
X : parts per Million : Fluorine19
F
O-N
Ph
F
3f

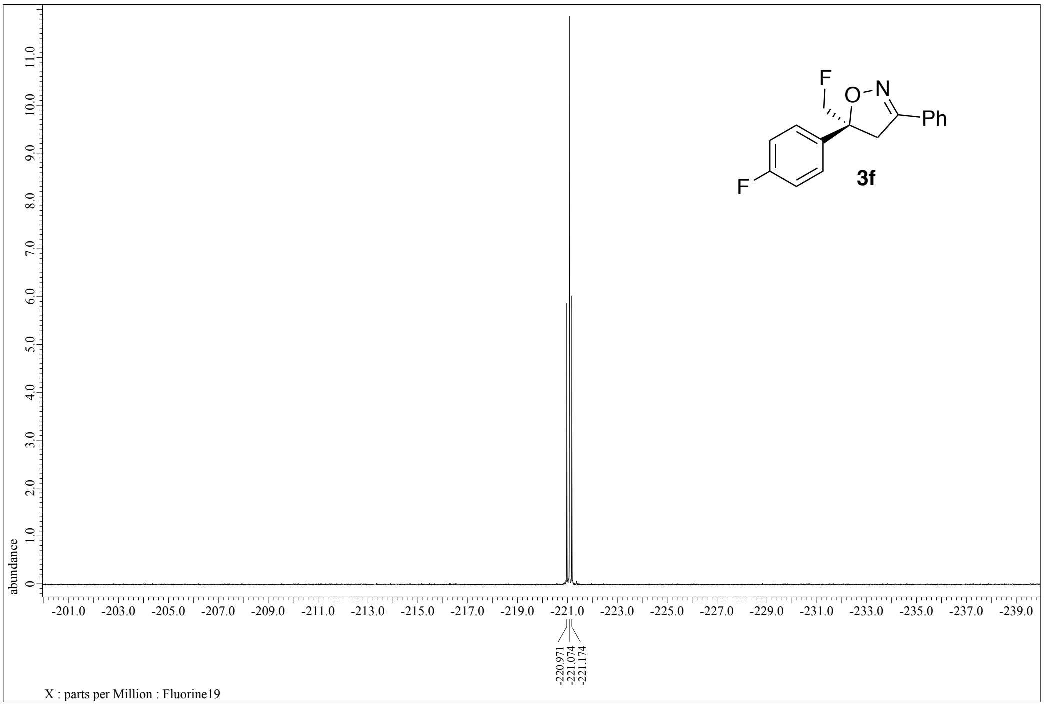
3f
abundance
X : parts per Million : Fluorine19
-220.971
-221.074
-221.174

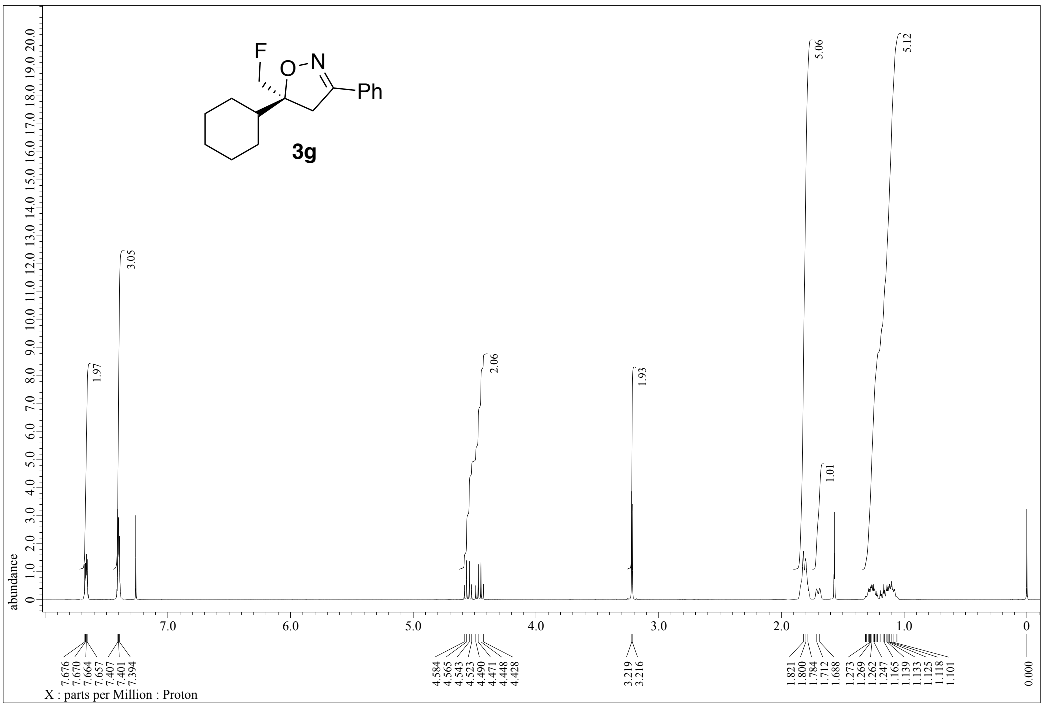
3g
abundance
X : parts per Million : Proton
1.97
3.05
2.06
1.93
5.06
1.01
5.12

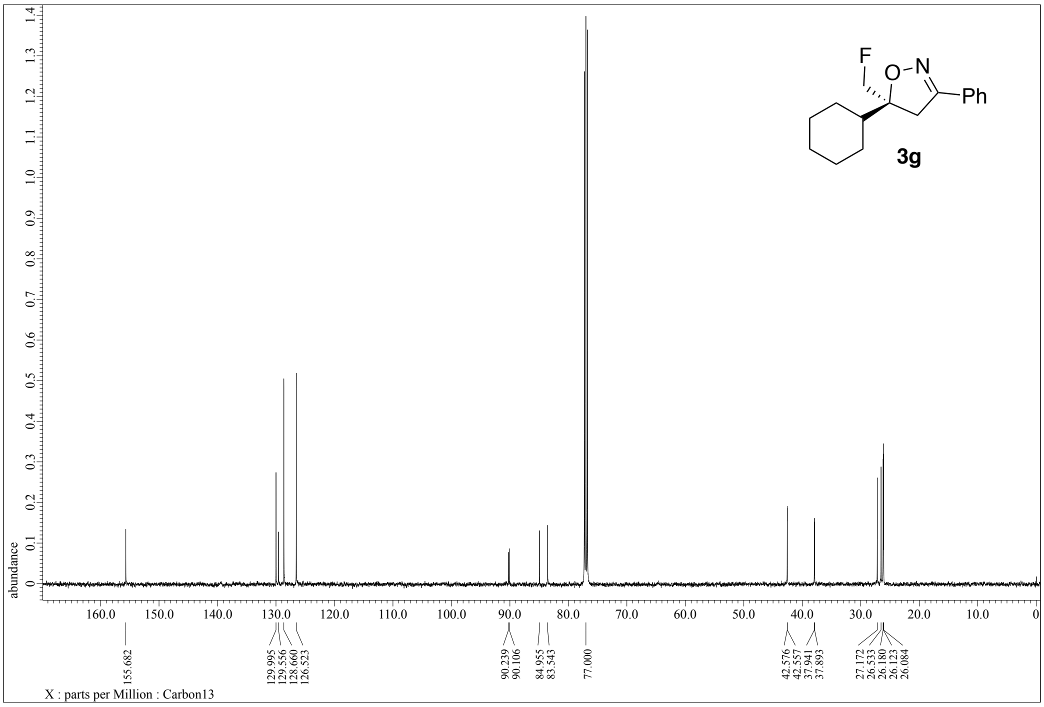

F
O–N
Ph
3g
abundance
X : parts per Million : Carbon13
155.682
129.995
129.556
128.660
126.523
90.239
90.106
84.955
83.543
77.000
42.576
42.557
37.941
37.893
27.172
26.533
26.180
26.123
26.084

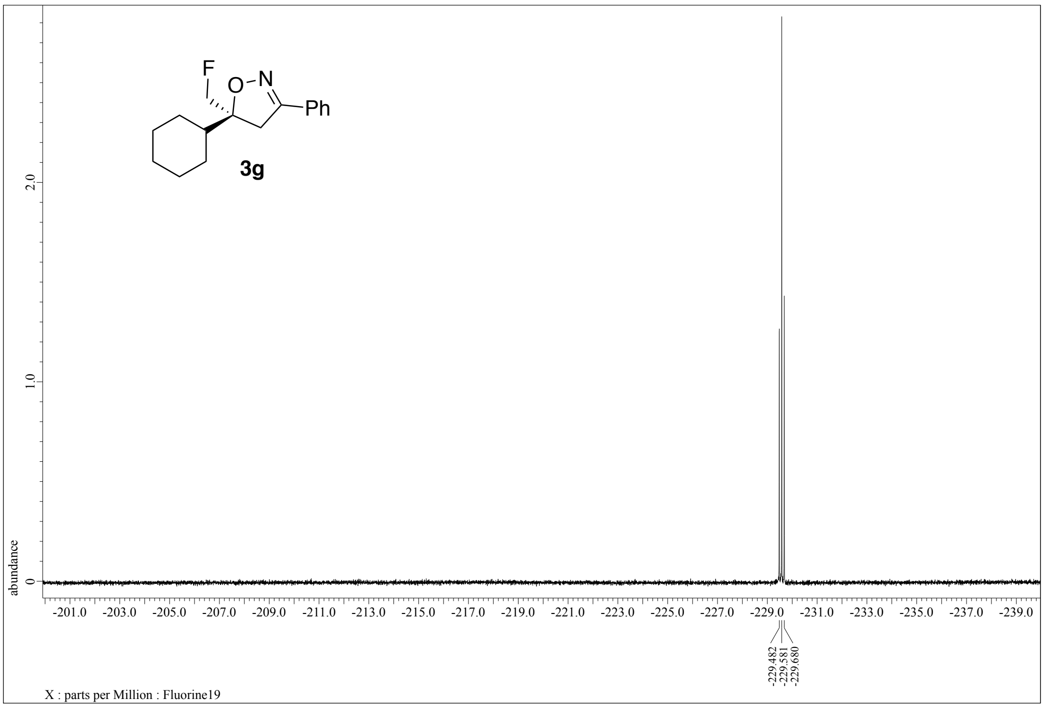

F
O–N
Ph
3g
abundance
X : parts per Million : Fluorine19
-229.482
-229.581
-229.680

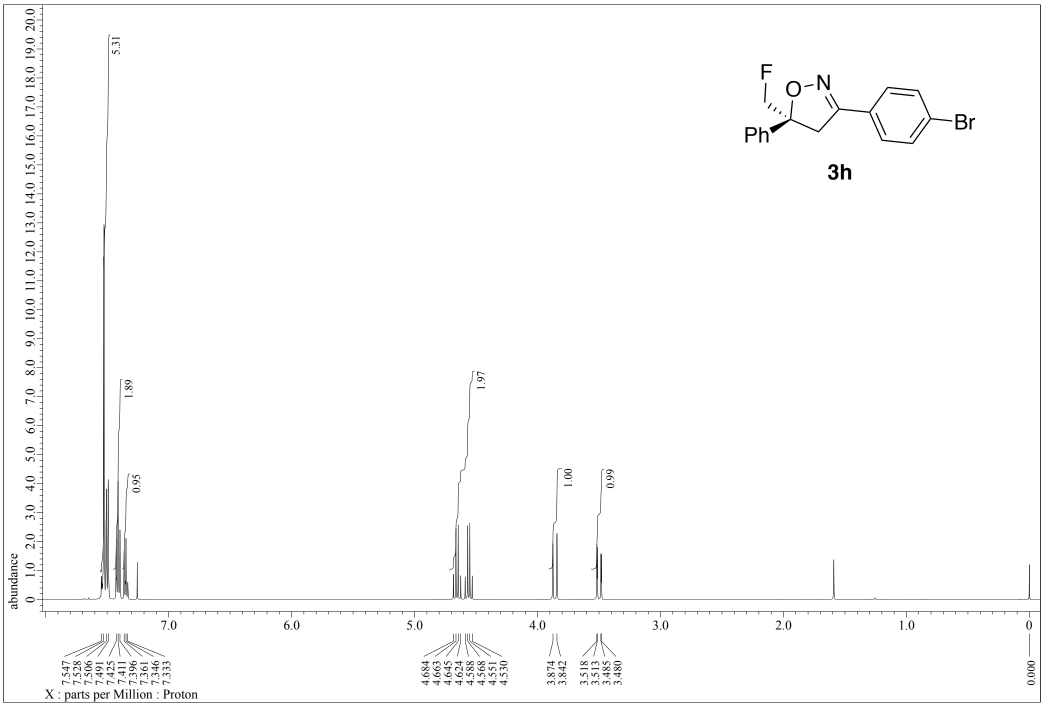
3h
X : parts per Million : Proton
abundance

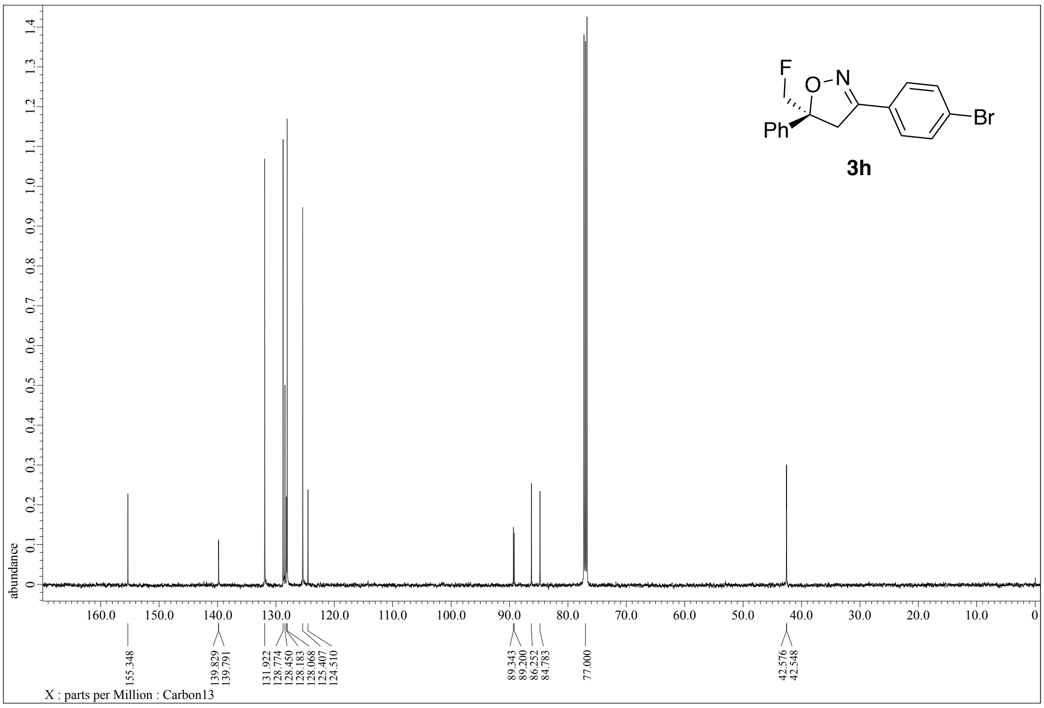
3h
X : parts per Million : Carbon13
abundance

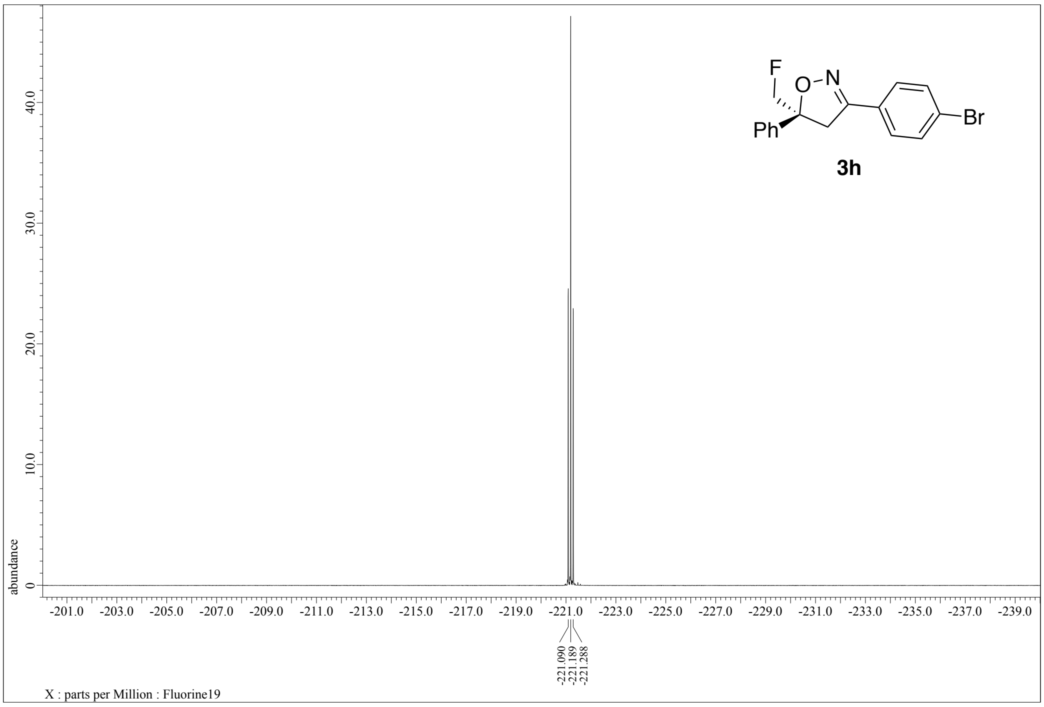
abundance
X : parts per Million : Fluorine19
-221.090
-221.189
-221.288
3h
Br

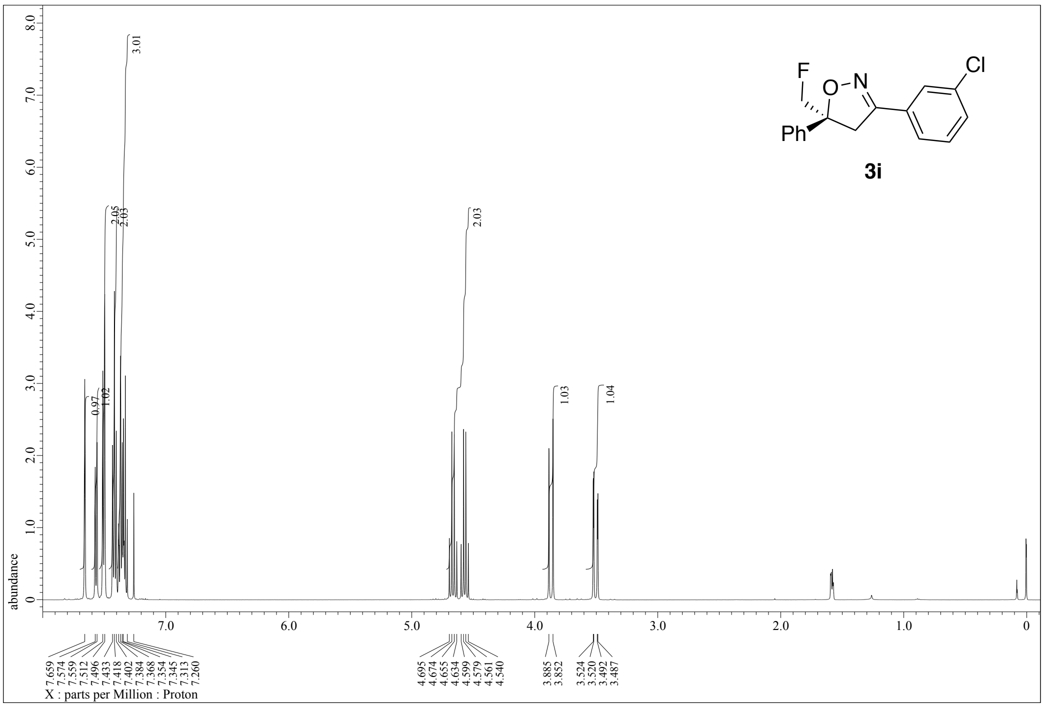
abundance
X : parts per Million : Proton
3i
Cl

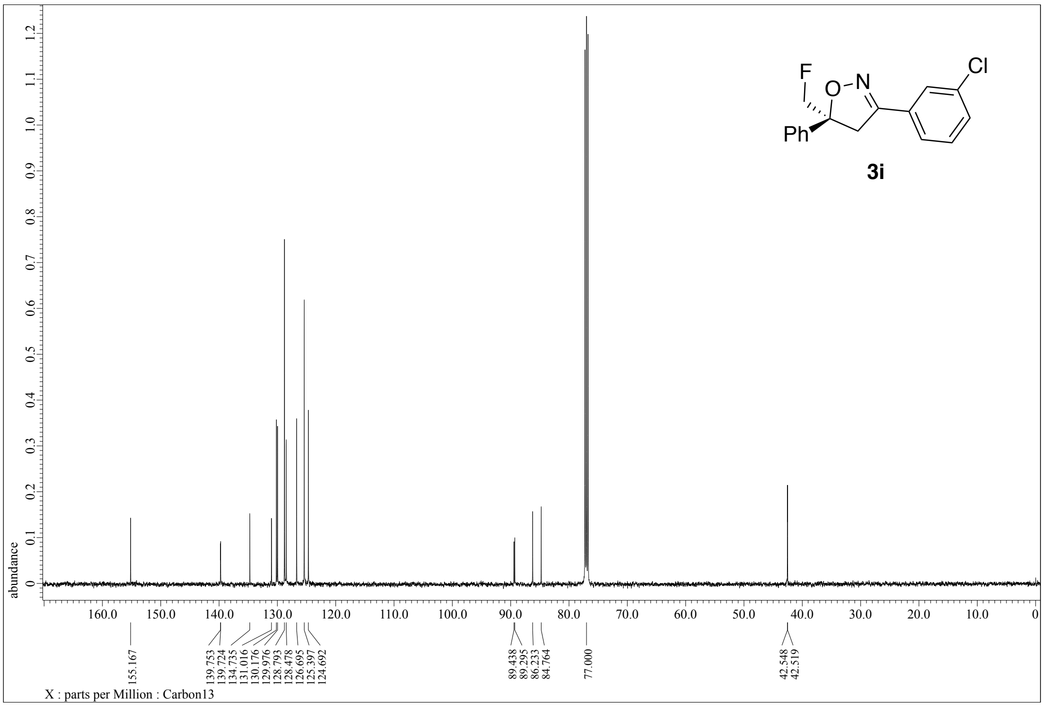

3i
abundance
X : parts per Million : Carbon13
155.167
139.753
139.724
134.735
131.016
130.176
129.976
128.793
128.478
126.695
125.397
124.692
89.438
89.295
86.233
84.764
77.000
42.548
42.519

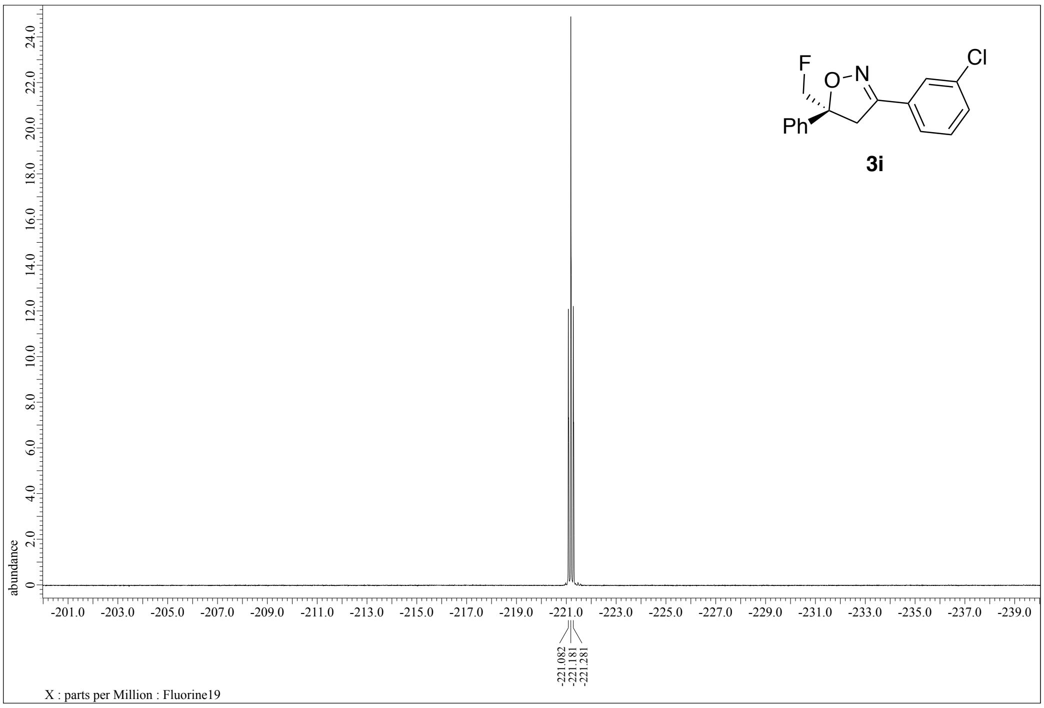

3i
abundance
X : parts per Million : Fluorine19
-221.082
-221.181
-221.281

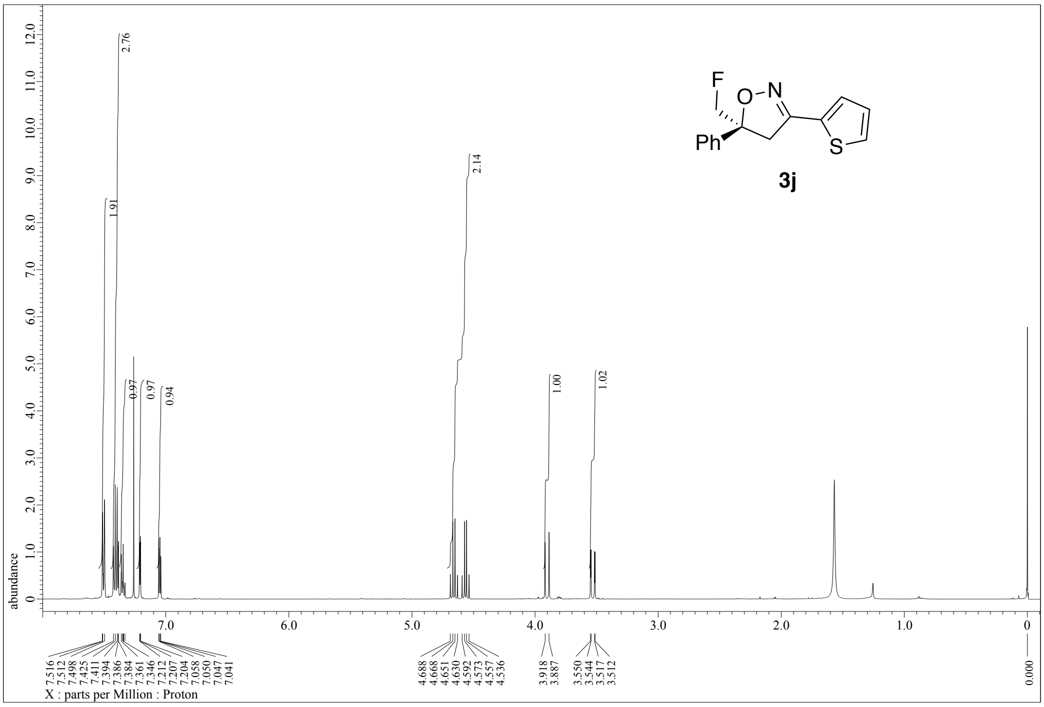
3j
X : parts per Million : Proton
abundance

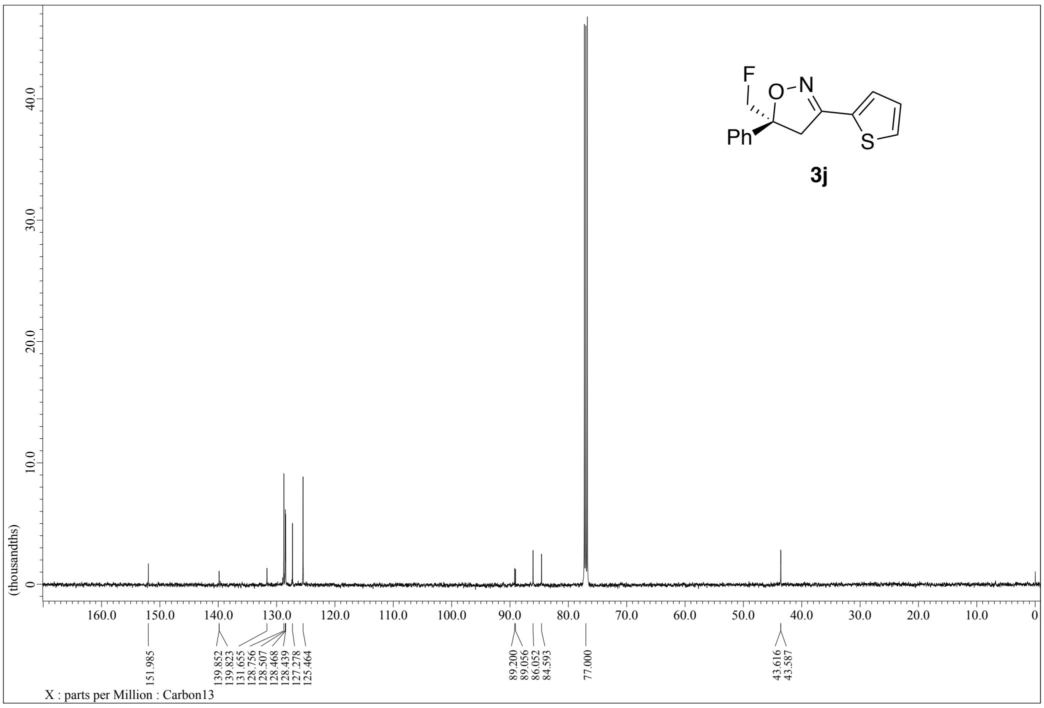
3j
X : parts per Million : Carbon13
(thousandths)

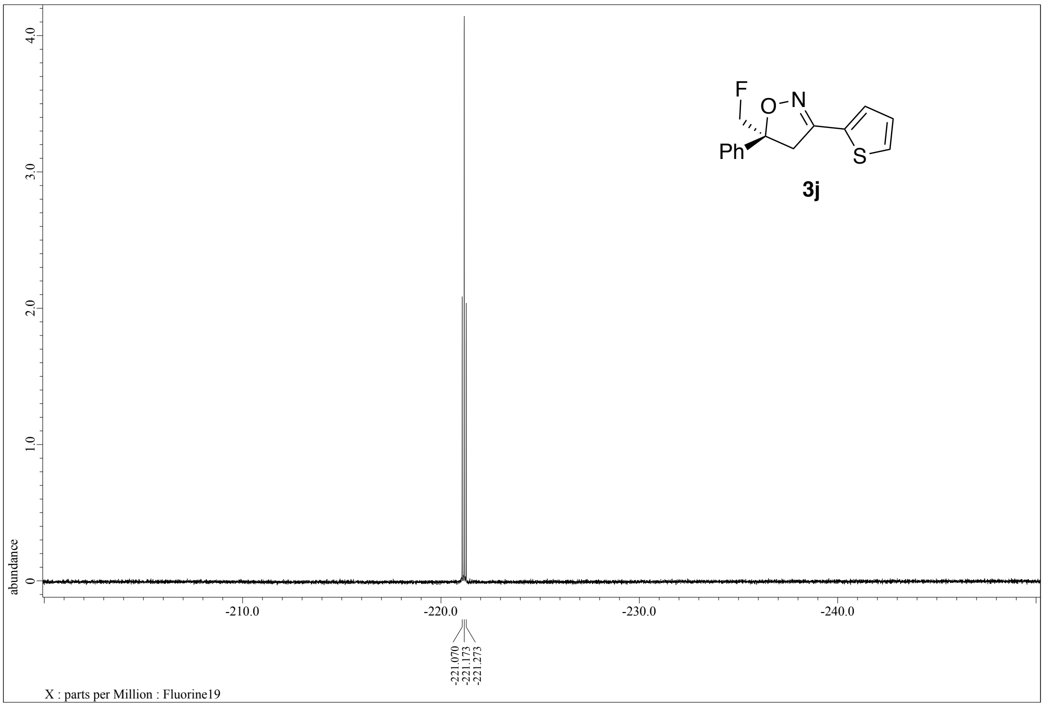

abundance
0
1.0
2.0
3.0
4.0
-210.0
-220.0
-230.0
-240.0
-221.070
-221.173
-221.273
X : parts per Million : Fluorine19
F
O
N
Ph
S
3j

2. HPLC data of compounds 3

Compound		Conditions	Retention time 1	Retention time 2
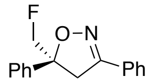	**3a**	IG-3, *n*-hexane/*i*-PrOH = 80/20, 1.0 mL/min	11.4 min (minor)	20.6 min (major)
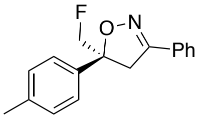	**3b**	IG-3, *n*-hexane/*i*-PrOH = 80/20, 1.0 mL/min	13.4 min (minor)	23.5 min (major)
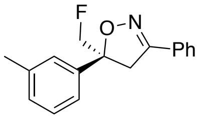	**3c**	IG-3, *n*-hexane/*i*-PrOH = 80/20, 1.0 mL/min	8.7 min (minor)	12.4 min (major)
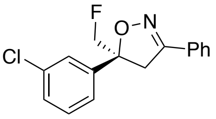	**3e**	IG-3, *n*-hexane/*i*-PrOH = 80/20, 1.0 mL/min	8.8 min (minor)	10.2 min (major)
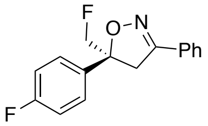	**3f**	IG-3, *n*-hexane/*i*-PrOH = 80/20, 1.0 mL/min	10.1 min (minor)	17.1 min (major)
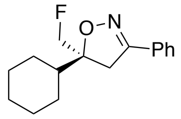	**3g**	IG-3, *n*-hexane/*i*-PrOH = 80/20, 1.0 mL/min	8.2 min (minor)	9.2 min (major)
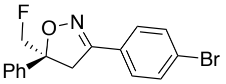	**3h**	IG-3, *n*-hexane/*i*-PrOH = 80/20, 1.0 mL/min	12.9 min (minor)	20.4 min (major)
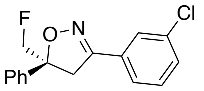	**3i**	IG-3, *n*-hexane/*i*-PrOH = 80/20, 1.0 mL/min	8.1 min (minor)	9.7 min (major)
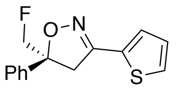	**3j**	IG-3, *n*-hexane/*i*-PrOH = 80/20, 1.0 mL/min	11.8 min (minor)	21.6 min (major)
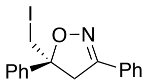	**8a**	ID-3, *n*-hexane/*i*-PrOH = 60/40, 1.0 mL/min	6.8 min (*R* isomer)	8.6 min (*S* isomer)[a]

[a] Tripathi, C.B.; Mukherjee, S. Catalytic Enantioselective Iodoetherification of Oximes. Angew. Chem. Int. Ed. **2013**, 52, 8450-8453.

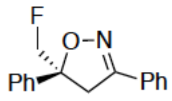

3a

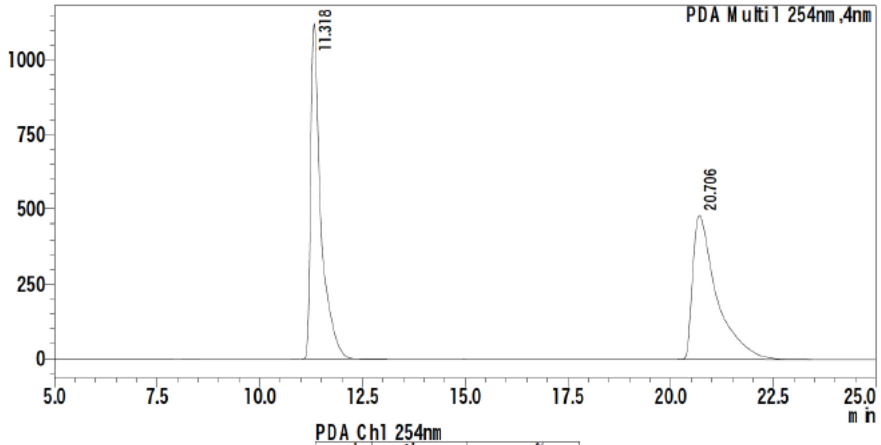

PDA Ch1 254nm

peak	time	area%
1	11.318	49.753
2	20.706	50.247
合計		100.000

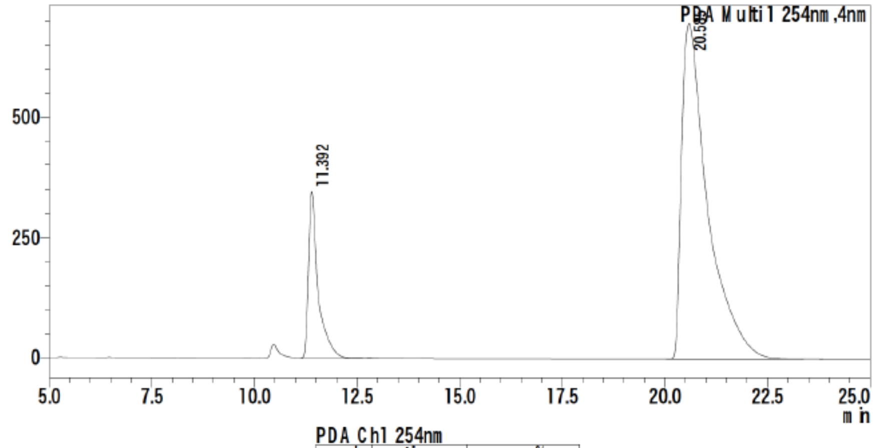

PDA Ch1 254nm

peak	time	area%
1	11.392	15.673
2	20.585	84.327
合計		100.000

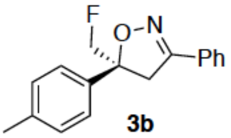

3b

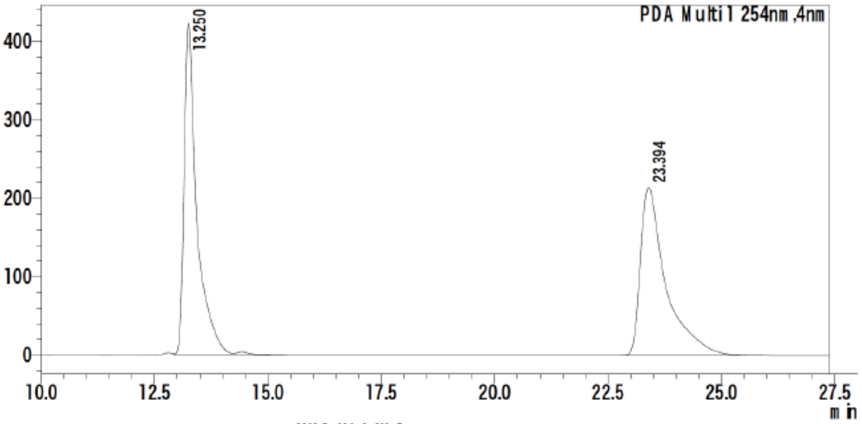

PDA Ch1 254nm

peak	time	area%
1	13.250	50.036
2	23.394	49.964
合計		100.000

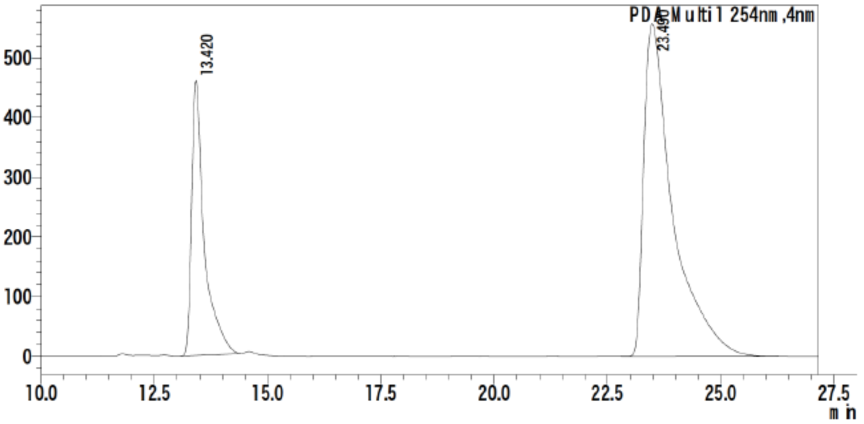

PDA Ch1 254nm

peak	time	area%
1	13.420	26.532
2	23.490	73.468
合計		100.000

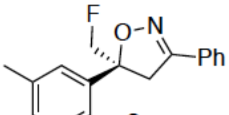

3c

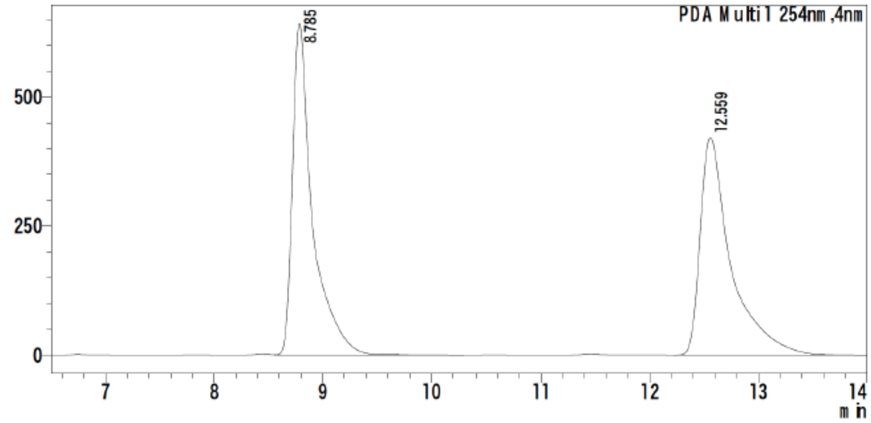

PDA Ch1 254nm

peak	time	area%
1	8.785	49.857
2	12.559	50.143
合計		100.000

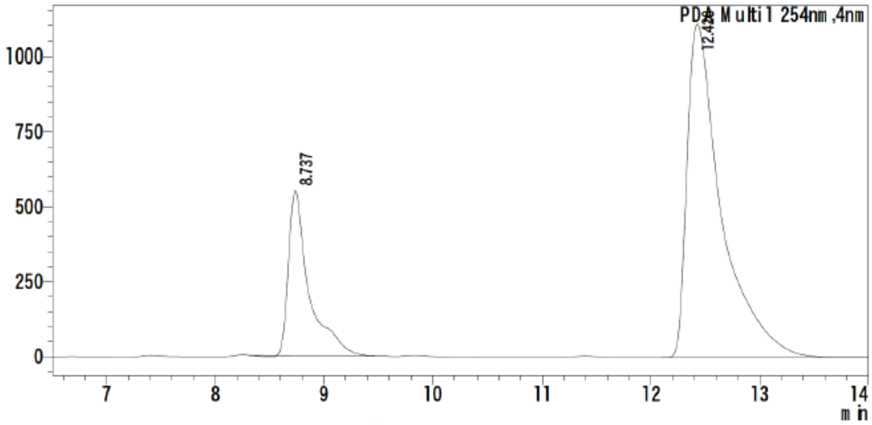

PDA Ch1 254nm

peak	time	area%
1	8.737	22.801
2	12.428	77.199
合計		100.000

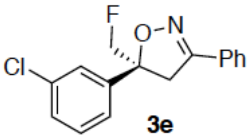

3e

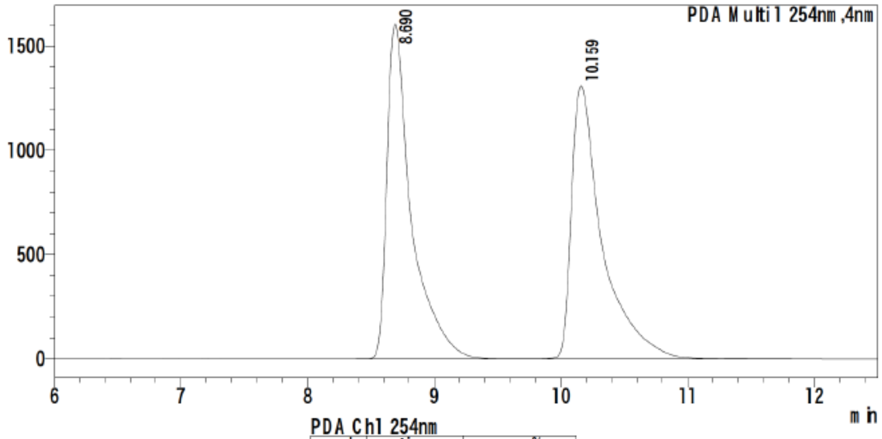

PDA Ch1 254nm

peak	time	area%
1	8.690	49.391
2	10.159	50.609
合計		100.000

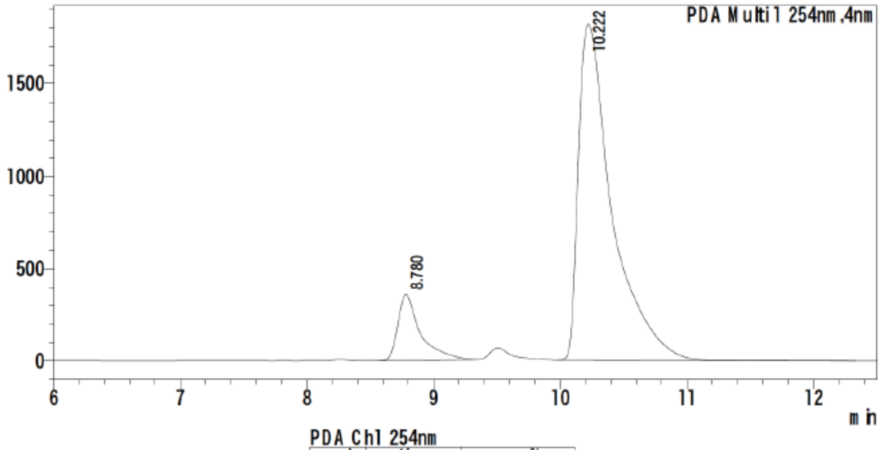

PDA Ch1 254nm

peak	time	area%
1	8.780	11.641
2	10.222	88.359
合計		100.000

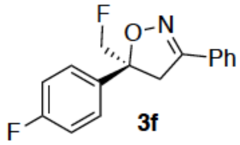

3f

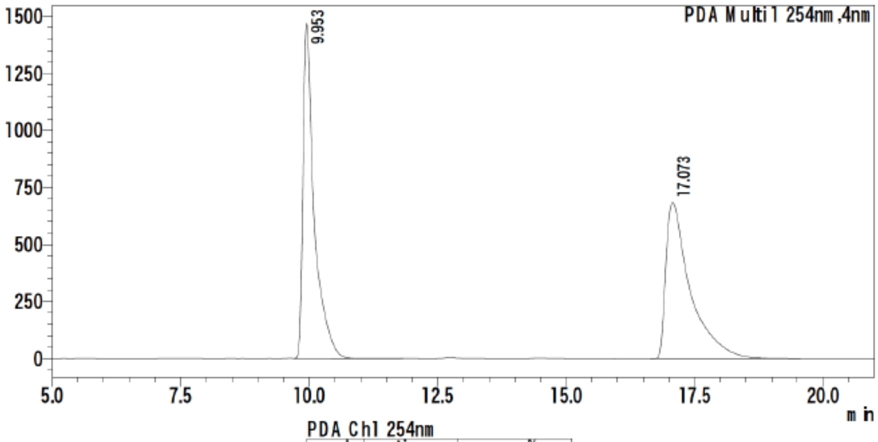

PDA Ch1 254nm

peak	time	area%
1	9.953	49.806
2	17.073	50.194
合計		100.000

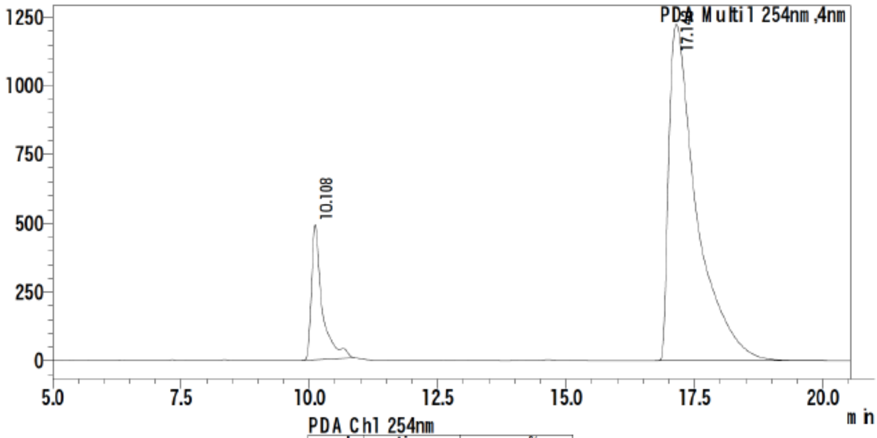

PDA Ch1 254nm

peak	time	area%
1	10.108	13.569
2	17.143	86.431
合計		100.000

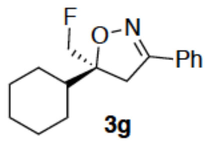

3g

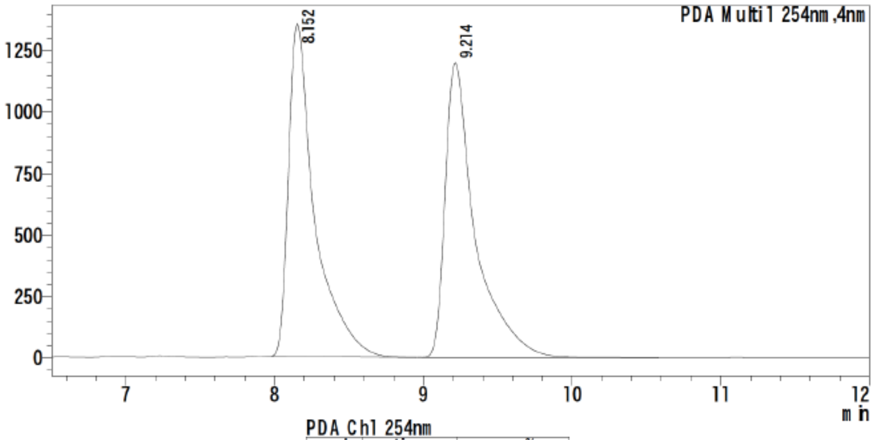

PDA Ch1 254nm

peak	time	area%
1	8.152	49.669
2	9.214	50.331
合計		100.000

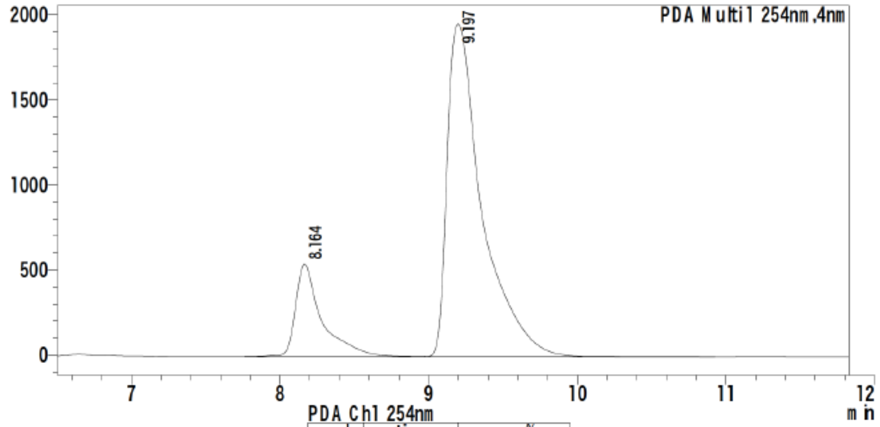

PDA Ch1 254nm

peak	time	area%
1	8.164	17.455
2	9.197	82.545
合計		100.000

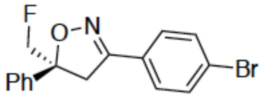

3h

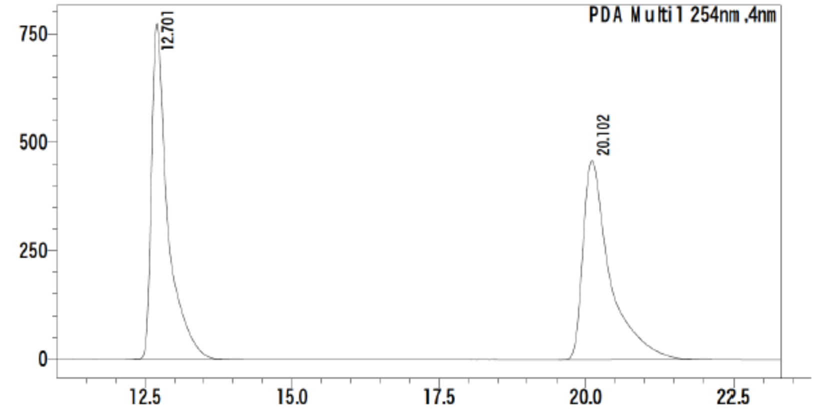

PDA Ch1 254nm

peak	time	area%
1	12.701	50.305
2	20.102	49.695
合計		100.000

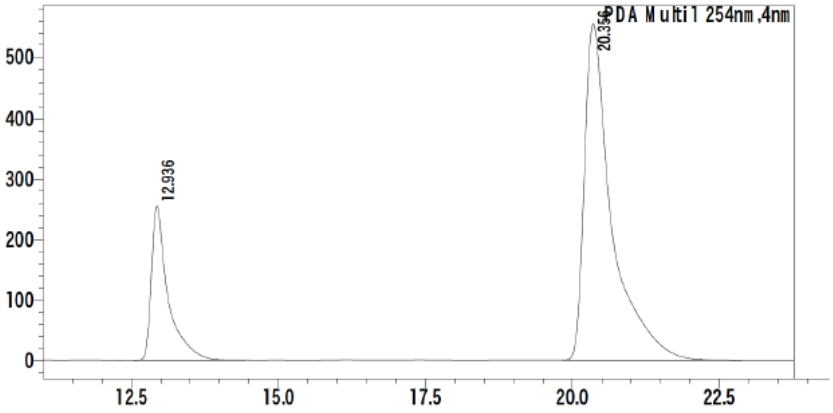

PDA Ch1 254nm

peak	time	area%
1	12.936	21.777
2	20.356	78.223
合計		100.000

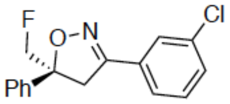

3i

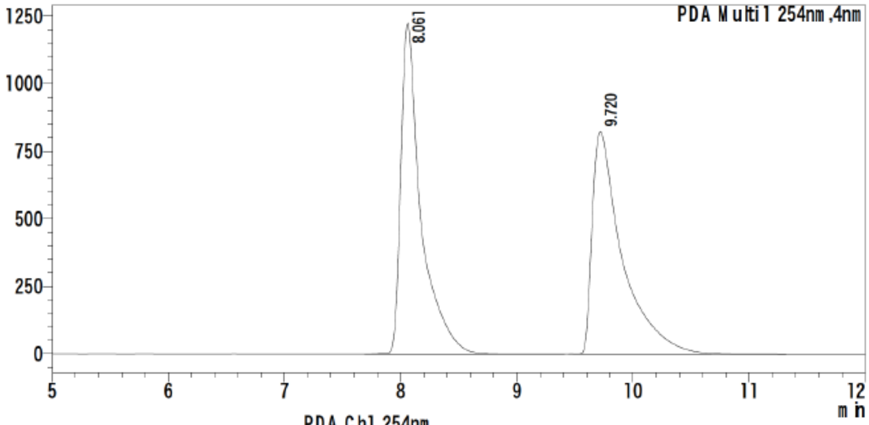

PDA Ch1 254nm

peak	time	area%
1	8.061	49.827
2	9.720	50.173
合計		100.000

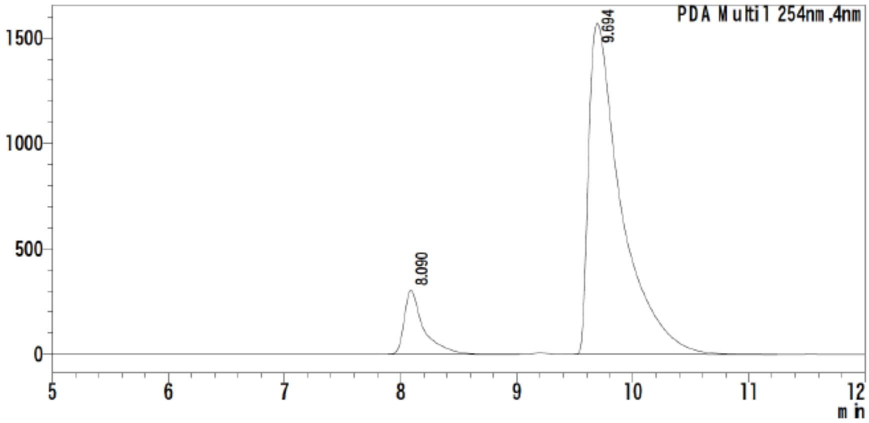

PDA Ch1 254nm

peak	time	area%
1	8.090	10.364
2	9.694	89.636
合計		100.000

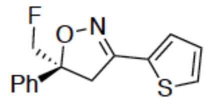

3j

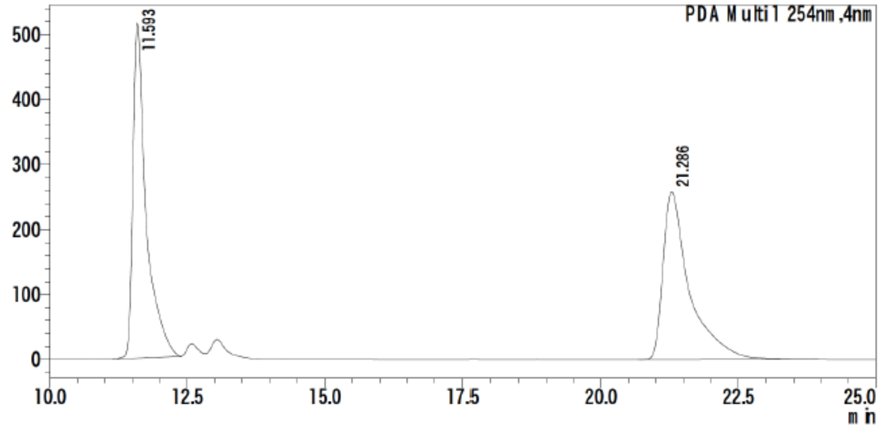

PDA Ch1 254nm

peak	time	area%
1	11.593	49.892
2	21.286	50.108
合計		100.000

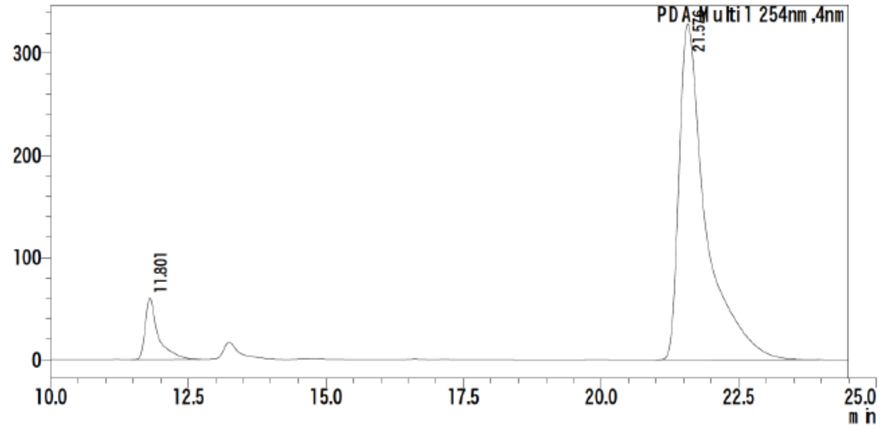

PDA Ch1 254nm

peak	time	area%
1	11.801	8.102
2	21.576	91.898
合計		100.000

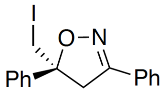

8a

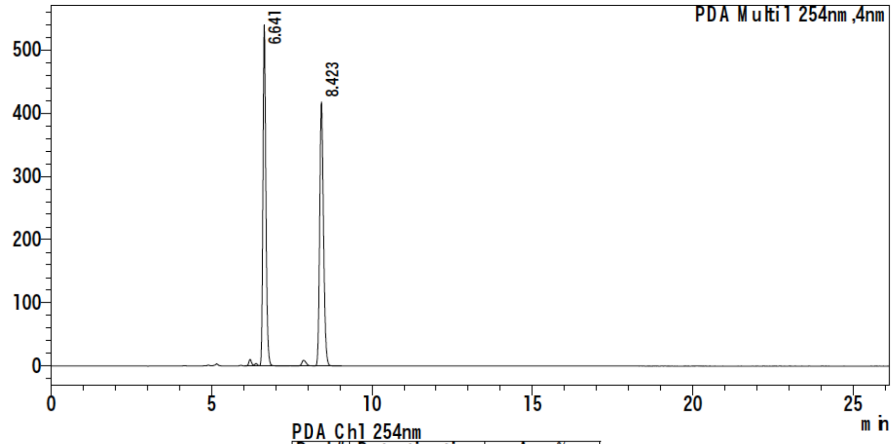

PDA Ch1 254nm

Peak#	Retention time	Area%
1	6.641	50.043
2	8.423	49.957

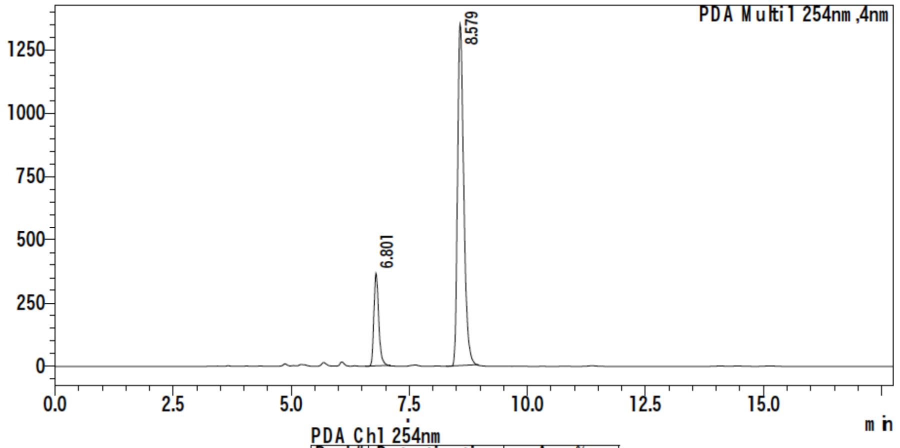

PDA Ch1 254nm

Peak#	Retention time	Area%
1	6.801	17.363
2	8.579	82.637
